# Pleiotropic Roles of NOTCH1 Signaling in the Loss of Maturational Arrest of Human Osteoarthritic Chondrocytes

**DOI:** 10.3390/ijms222112012

**Published:** 2021-11-05

**Authors:** Manuela Minguzzi, Veronica Panichi, Stefania D’Adamo, Silvia Cetrullo, Luca Cattini, Flavio Flamigni, Erminia Mariani, Rosa Maria Borzì

**Affiliations:** 1 Dipartimento di Scienze Mediche e Chirurgiche, Università di Bologna, 40138 Bologna, Italy; manuela.minguzzi@gmail.com (M.M.); stefania.dadamo2@unibo.it (S.D.); erminia.mariani@ior.it (E.M.); 2 Dipartimento di Scienze Biomediche e Neuromotorie, Università di Bologna, 40138 Bologna, Italy; veronica.panichi2@unibo.it (V.P.); silvia.cetrullo@unibo.it (S.C.); flavio.flamigni@unibo.it (F.F.); 3 Laboratorio di Immunoreumatologia e Rigenerazione Tissutale, IRCCS Istituto Ortopedico Rizzoli, 40136 Bologna, Italy; luca.cattini@ior.it

**Keywords:** osteoarthritis, chondrocytes, hypertrophy, remodeling, angiogenesis

## Abstract

Notch signaling has been identified as a critical regulator of cartilage development and homeostasis. Its pivotal role was established by both several joint specific Notch signaling loss of function mouse models and transient or sustained overexpression. NOTCH1 is the most abundantly expressed NOTCH receptors in normal cartilage and its expression increases in osteoarthritis (OA), when chondrocytes exit from their healthy “maturation arrested state” and resume their natural route of proliferation, hypertrophy, and terminal differentiation. The latter are hallmarks of OA that are easily evaluated in vitro in 2-D or 3-D culture models. The aim of our study was to investigate the effect of NOTCH1 knockdown on proliferation (cell count and Picogreen mediated DNA quantification), cell cycle (flow cytometry), hypertrophy (gene and protein expression of key markers such as RUNX2 and MMP-13), and terminal differentiation (viability measured in 3-D cultures by luminescence assay) of human OA chondrocytes. NOTCH1 silencing of OA chondrocytes yielded a healthier phenotype in both 2-D (reduced proliferation) and 3-D with evidence of decreased hypertrophy (reduced expression of RUNX2 and MMP-13) and terminal differentiation (increased viability). This demonstrates that NOTCH1 is a convenient therapeutic target to attenuate OA progression.

## 1. Introduction

Osteoarthritis (OA) is the leading cause of chronic disability in the elderly, with worldwide estimates showing that 9.6% of men and 18.0% of women over 60 years old suffer for symptomatic OA [1]. Therefore, OA represents a huge problem for the quality of life of millions of individuals and for the national healthcare systems. Established OA is associated with the progressive derangement of articular cartilage structure and function and is maintained by positive feedback loops with the involvement of subchondral bone and synovial tissue [2]. However, at its onset most often OA is an articular cartilage disease caused by the failure of the homeostatic mechanisms that actively maintain chondrocytes in a differentiation arrested state [3]. Indeed, chondrocytes are post-mitotic cells that in healthy cartilage do not proliferate and are only in charge of a very limited turnover of extracellular matrix (ECM) [4]. It has become increasingly evident that degeneration of articular cartilage is sustained by multiple inflammatory loops that can be targeted in order to restore the functionality of the tissue or at least to delay OA progression [5].

Notch signaling has been identified as a critical regulator of cartilage development and homeostasis with an on/off expression in the different phases of chondrogenesis as resumed in the growth plate [6]. As detailed elsewhere, the activation of this pathway occurs after triggering the receptor with a ligand [7]. The pathway requires proteolytic cleavages of the internal portion of the transmembrane receptor, which translocates to the nucleus to act as a transcription coactivator on target genes, thereby being renamed as Notch intracellular domain (NICD). NICD binds to RBPJ (recombination signal-binding protein for Ig region) and Maml (mastermind-like) in order to start transcription [7]. Although there is occurrence of multiple crosstalks with other pathways as detailed in [8], Notch activation directly leads to the induction of several target genes, among which HES1 (hairy and enhancer of split-1) has been recognized as relevant in OA pathogenesis [9].

Expression of members of the Notch pathway occurs in early differentiation phases of skeletal development, such as mesenchymal progenitor condensation [10]. Likewise, functional genomic studies carried out in mice indicated that proper functioning of the pathway in postnatal chondrocytes is required for the correct cartilage differentiation as well as for the expression of ECM genes (col2, aggrecan), transcription factors (Sox9), and ECM degrading enzymes (adamts 4, adamts 5, and mmp-13) [11]. Later on, a subsequent time specific expression of Notch1 and Notch2 drives the progression from the proliferating to the pre-hypertrophic and hypertrophic chondrocyte phenotype in the context of endochondral ossification in the growth plate [6].

Although transient (growth plate) and stable (articular) cartilages are different tissues from an anatomical and morphogenetic point of view, steps occurring in the former have represented a reference to understand OA pathogenesis. This represents the so-called EVO-DEVO approach [4], i.e., a pathogenetic model whereby functional changes occurring in OA in adulthood in many species (EVO) are interpreted as an attempt to recapitulate developmental programs (DEVO) to contrast structural changes. The expression of NOTCH receptors and ligands in healthy articular cartilage has been reported several years ago [12] as well as changes due to OA. Both NOTCH1 and NOTCH2 were found expressed in human cartilage particularly in the superficial zone, but major changes in OA are related to NOTCH1, whose increased expression in OA cartilage, particularly in the so-called “clusters”, suggests its involvement in the abnormal cell activation and differentiation process of OA chondrocytes [13,14]. Indeed, NOTCH1 and NOTCH2 move to the nucleus in chondrocytes of developing growth plate cartilage and drive endochondral ossification via RPBJ signaling and are also found increased in murine and human OA [15]. Karlsson et al. showed the dramatic increase of the cells positive for NOTCH1, Jagged, and HES in OA compared to healthy cartilage [16]. A more recent study reported an increase in NOTCH1, Jagged-1, HES1, and NICD1 in human OA, a pattern also reproduced in a murine OA model [17]: the destabilization of the medial meniscus (DMM), the method of choice for surgically induced OA in mice that reproduces the slow onset and progression of human OA [18,19]. Interestingly, Jagged-1, the pivotal trigger of NOTCH1 activation is a NF-κB target gene [20] and resulted upregulated at early (2 weeks) stages of DMM [19] as shown in a matched comparison of nine microarrays carried out in similar conditions [21]. This suggests an early and pivotal involvement of the NOTCH pathway in OA development as well as occurrence of crosstalks with other master signaling pathways in OA.

Gain of function mouse models for the Notch pathway have shown an OA-like phenotype [15,22,23]. On the other hand, a chondroprotective role of “housekeeping” NOTCH levels is consistent with worsening of the disease following Notch1 inactivation in surgical mouse OA models [17,24]. Therefore, in human articular cartilage, the NOTCH pathway might have a dual role both in cartilage homeostasis maintenance and OA development. NOTCH1 and its target genes are overexpressed in OA compared to healthy articular cartilage [14]. Conversely, the prevention of pathway activation by γ-secretase inhibitors leads to reduced expression of ECM catabolic factors in in vitro cultured chondrocytes [15,22,25].

A recent report has shed light on the signaling network that links NOTCH1 with chondrocyte proliferation, differentiation, and MMP-13 expression via RUNX2 [26] and modulation with γ secretase inhibition (via *N*-[*N*-(3,5-Difluorophenacetyl)-l-alanyl]-S-phenylglycine t-butyl ester or DAPT) or Jagged induction.

Despite the large amount of information reported above, further investigation is needed to shed light on the role of NOTCH1 role in human OA. In particular, no literature data are available about the effects of NOTCH inactivation on human primary OA chondrocytes. As described above, the pathway is overexpressed in OA compared to healthy chondrocytes, and a nonspecific DAPT-dependent NOTCH pathway inactivation exerts chondroprotective and anabolic effects in human OA cartilage explants [27]. Therefore, tuning NOTCH signaling down to a homeostatic level may represent one of the constraints that prevent progression of the differentiation of articular chondrocytes toward hypertrophy.

The aim of our work has been to characterize the effects of NOTCH1 silencing on major features of OA chondrocytes (increased proliferation and loss of maturational arrest driven by increased extracellular remodeling) in suitable culture models. Given the pleiotropic effects of NOTCH that are peculiar to the differentiation stage of the cells, we undertook the present study to characterize the effects of its silencing on proliferation and differentiation in vitro, exploiting both 2-D and 3-D culture models, established with primary human OA chondrocytes, in the perspective of elaborating a potential therapeutic strategy for OA management. Some preliminary findings presented in this manuscript have been already presented at an OARSI Congress in 2018 [28].

## 2. Results

### 2.1. NOTCH1 Expression Is Higher in 2-D Compared to 3-D Chondrocyte Culture

**Figure 1:**
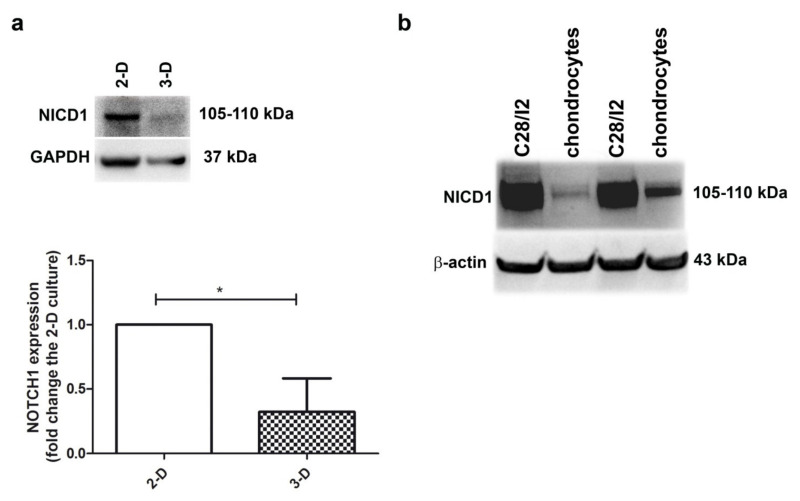
Higher NOTCH1 expression is evident in proliferating chondrocyte cultures. (**a**) To assess NOTCH1 expression and modulation in human chondrocyte cultures, the active form of the receptor was evaluated in monolayer culture and in micromasses at 1 week maturation. GAPDH was used as a loading control. A representative example and a cumulative evaluation performed with samples from three different patients. * *p* < 0.05. (**b**) Western blot analysis of NOTCH1 expression in C28/I2 cells compared to primary chondrocytes, with β-actin as a loading control.

Figure 1a shows that the level of NOTCH1 (as assessed by evaluating the intensity of the NICD1 band at 105–110 kDa) was higher in 2-D (monolayer) compared to 3-D (micromass) chondrocyte cultures (*p* < 0.05, *n* = 3), the latter representing a culture model where chondrocytes stop proliferation and recover their differentiated status, with progression to hypertrophy and terminal differentiation over time [29]. On the other hand, monolayer chondrocyte cultures maintain a small fraction of proliferating cells, although its percentage is further reduced at high density [30]. Thus, the different NOTCH1 level may be related to the proliferative status. The relationship between NOTCH1 expression level and the percentage of cells in mitosis is further highlighted in Figure 1b and Supplementary Figures S2 and S3: NOTCH1 expression is much higher in the immortalized chondrocyte cell line C28/I2 compared to primary chondrocytes, with the former showing about 40% cells in the S-G_2_M cell cycle phases [31] and the latter about 5% [30].

The level of cleaved NOTCH1 (Val1744) was also investigated in both primary chondrocytes and C28/I2 cells upon stimulation with EDTA [32] or IL-1 [9]. Supplementary Figure S4 shows that the level of this antigen is almost undetectable in primary chondrocytes.

With regard to NOTCH1 proteolytic processing, it is well known that NOTCH1 cleaved at the level of Val1744 is the result of sequential cleavages of the membrane bound NOTCH1 [8]: (1) S1: furin mediated cleavage to obtain a 120 kDa peptide, (2) S2: ADAM10 or 17 mediated cleavage to obtain a 115 kDa, and (3) S3: γ-secretase mediated cleavage to obtain the 110 kDa NICD1 peptide that exposes Val1744 epitope [32].

The NICD1 band presented in our Western blot has a molecular weight of 105–110, therefore, it likely corresponds to the NICD1 fragment, that possibly becomes suddenly post-translational modified with masking/degradation of the epitope, so that the signal of anti-cleaved (Val1744) NOTCH1 is hardly detectable at least in primary chondrocytes (Supplementary Figure S4).

Despite the marked difference in band intensity, a similar pattern of anti-cleaved (Val1744) NOTCH1 is observable in both C28/I2 and primary chondrocytes, with a fraction of the signal under 1 h EDTA treatment showing higher molecular weight in both cell types. This possibly indicates a fraction of the molecules that undergo marked phosphorylation with a massive apparent shift (5–10 kDa) [33] in molecular weight. Phosphorylation, the “fulcrum of NOTCH1 signaling [34]”, then increases activity of NICD1, that is however immediately degraded. Taken together, the findings presented in Figure 1b and Supplementary Figure S4, we can conclude that there is much less active NOTCH1 in primary chondrocytes, i.e., cleaved NICD1 able to enter the nucleus and regulate NOTCH1 target genes in a canonical (RBPJ dependent) way. In addition, the protease rich cellular environment of chondrocytes may concur to rapidly terminate the pathway.

### 2.2. NOTCH1 Transient Silencing Is Efficient at Both Protein and RNA Level

**Figure 2:**
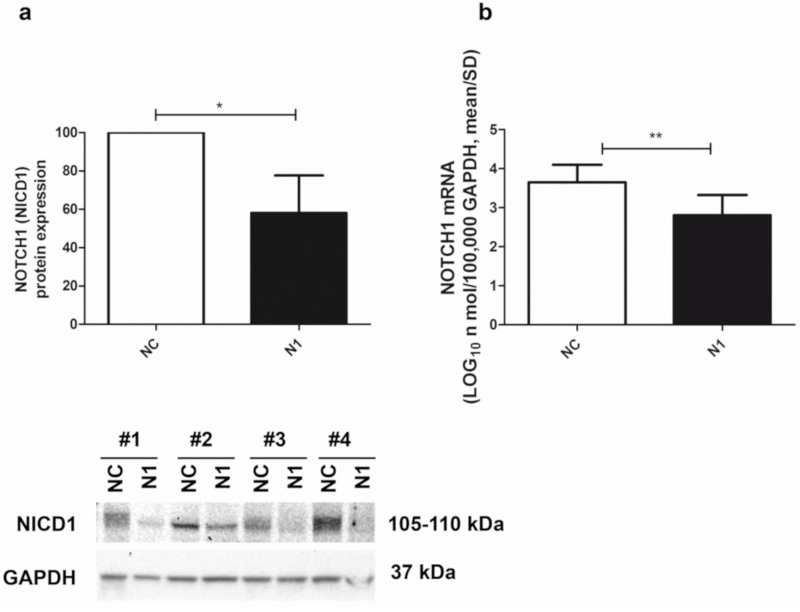
Efficiency of NOTCH1 silencing. (**a**) Cumulative Western blot evaluation of NOTCH1 KD in four different samples (Western blot shown below) as evaluated by assessment of the 110 kDa NICD1 active form at 48 h after transfection (mean ± SD, *n* = 4) with GAPDH used as a loading control; * *p* < 0.05. (**b**) NOTCH1 KD in five different samples assessed by real time RT-PCR (mean ± SD, *n* = 5). NC—control; N1—NOTCH1 silenced chondrocytes; ** *p* < 0.01.

The siRNA-mediated NOTCH1 silencing of the primary chondrocyte cultures was quite efficient, as determined by both Western blot and real time RT-PCR. At the protein level, assessment in Western blot of the intensity of the NOTCH1 intracellular and transcriptionally active domain (NICD1 fragment of about 110 kDa) pointed at a 58 ± 20 (mean ± SD, *n* = 4) percentage reduction in NOTCH1 KD cells (N1) compared to the level in the control siRNA (NC) chondrocytes (Figure 2, left image). At the RNA level, assessment in real time RT-PCR of NOTCH1 expression in NOTCH1 KD chondrocytes at 48 h post transfection pointed at a 82 ± 14 (*p* = 0.0023, mean ± SD, *n* = 5) percentage reduction compared to the level in the control siRNA cells (Figure 2, right image). The difference was statistically significant.

The earlier effects of the siRNA on NOTCH1 transcripts are consistent with the higher knockdown efficiency as assessed by mRNA compared to the protein quantification. This is possibly because in the case of abundantly expressed proteins such as NOTCH1 the cells need time to get rid of it according to the physiological turnover of the siRNA target. On the other hand, the downstream experimental design of our work relied on the stability of the silencing effects. For this purpose, it has been reported that rather than on intracellular siRNA half-life, the duration of gene silencing is mostly dependent on dilution effects due to cell division, with differences according to the type of cells: rapidly dividing or slowly dividing (such as chondrocytes), with a duration of at least 3 weeks in the latter [35].

### 2.3. NOTCH1 KD Determines Biphasic Effects on Cell Proliferation

Compared to the effects on NOTCH1 RNA, the delayed kinetic of NOTCH1 protein reduction was also reflected in the different proliferative behavior of cells after NOTCH1 silencing at early or late time points.

**Figure 3:**
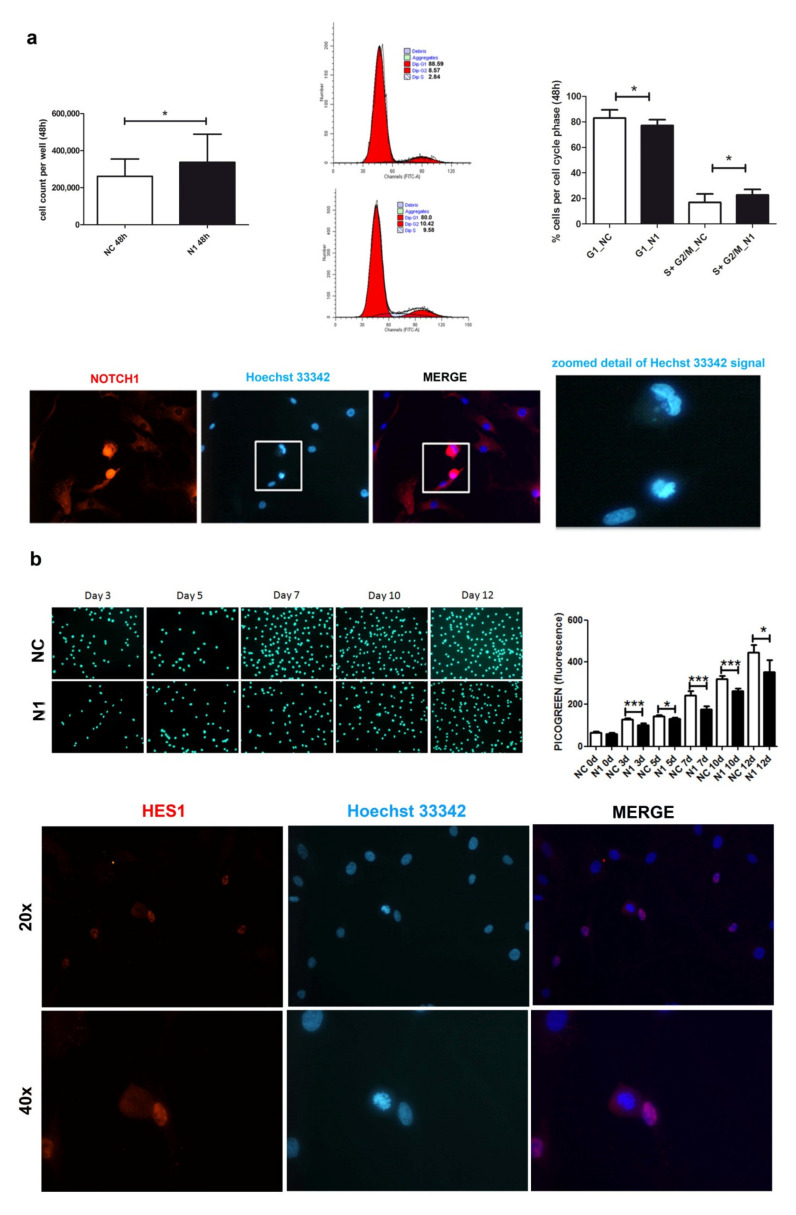
Effects of NOTCH1 silencing on cell proliferation. (**a**) Early effects (at 48 h) of increased cell proliferation in different NOTCH1 KD primary chondrocyte cultures (*n* = 7) in keeping with statistically significant increased percentage of cells in the S-G_2_M fraction as shown by one representative sample and cumulative data (*n* = 4); * *p* < 0.05. Immunofluorescence indicates the close NOTCH1/DNA interaction during mitosis. Bottom: NOTCH1 immunofluorescence in chondrocytes at 72 h post control siRNA transfection. NOTCH1 staining (red), nuclei (blue), and merged images obtained with a NIKON Eclipse 90i fluorescence microscope equipped with 20 × objective lens. The white square indicates a detail that has been further zoomed and represented in the right image to indicate that the cells with strong NOTCH1 signal are caught during mitosis based on condensed chromatin. (**b**) Late effects (plating the cells recovered at 48 h post-transfection and assessment at days 0, 3, 5, 7, 10, 12) of delayed cell proliferation in NOTCH1 KD chondrocytes. Upper image shows the signal of the highly specific Picogreen staining of the nuclei of control cells (upper row) or NOTCH1 silenced cells (lower row). The right graph represents the mean ± SD of the fluorescence intensity obtained by scanning the bottom of the quintuplicate wells; * *p* < 0.05, *** *p <* 0.001. Bottom: HES1 immunofluorescence in chondrocytes at 72 h post control siRNA transfection. HES1 staining (red), nuclei (blue), and merged images obtained with a NIKON Eclipse 90i fluorescence microscope equipped with either 20× objective lens (with gain 1.2×, upper row) or 40x (with gain 1.2×, lower row).

At 48 h post transfection (Figure 3a), NOTCH1 silencing gave an advantage in cell proliferation. Cell count of different primary chondrocyte cultures indicated a higher recovery of NOTCH1 KD cells (*p* = 0.033, *n* = 7), in keeping with a significantly lower percentage of cells in the G1 phase and higher percentage of cells in the S-G_2_M phase (*p* = 0.0331, *n* = 4).

Immunofluorescence indicated a stronger and mainly nuclear NOTCH1 staining in the cells during mitosis, suggesting a close interaction of NOTCH1 with DNA compared to those in interphase (Figure 3a).

Possibly because of its involvement in DNA replication [36], at a later analysis, NOTCH1 silencing showed opposite consequences in cell proliferation, as shown in Figure 3b that presents Picogreen (i.e., a specific dye for dsDNA) staining relative to one out of three different experiments performed, along with the quantitative assessment of fluorescence performed in quintuplicate that shows decreased cell proliferation of NOTCH1 KD cells. The right graph shows the cumulative data represented as mean ± standard deviation. The reason for the impaired cell proliferation may correspond to a reduced expression of HES1, the main NOTCH target gene. HES1 localizes in actively dividing cells suggesting an active role in controlling cell cycle progression, namely G_1_/S transition as also reported in other cell types [37] and in the output of the research for pathways in the Reactome pathway analyzer, (https://reactome.org/PathwayBrowser/#/DTAB=AN&ANALYSIS=MjAyMTA2MTUxNDQ2NTJfMTU%3D&FLG=HES1, accessed on 25 march 2021).

### 2.4. NOTCH1 KD Determines a Delayed Differentiation in 3-D Culture 

#### 2.4.1. Reduced Transcription of Differentiation Related Genes

The data provided above about the delayed but persistent clearance of the protein were confirmed in the evaluation of the effects of NOTCH1 silencing in differentiation recapitulated in 3-D cultures [38], as a model whereby evaluating correlated phenomena in differentiation progression from hypertrophy to terminal differentiation, including hypertrophy, catabolic markers, and chondrocyte viability. The 3-D cultures were established at 48 h after transfection with either control or NOTCH1 silenced chondrocytes, so that the amounts of siRNA in the cells were not diluted because of division [35] and collected at 1, 2, and 3 weeks. Data shown in Supplementary Figure S6 show the persistence of NOTCH1 silencing over time. To analyze the effects on transcription we focused on micromasses at 1 week maturation. We selected this time point in order to avoid biases connected with the reduced viability at later time points due to recapitulation of terminal differentiation in these 3-D cultures [29].

**Figure 4:**
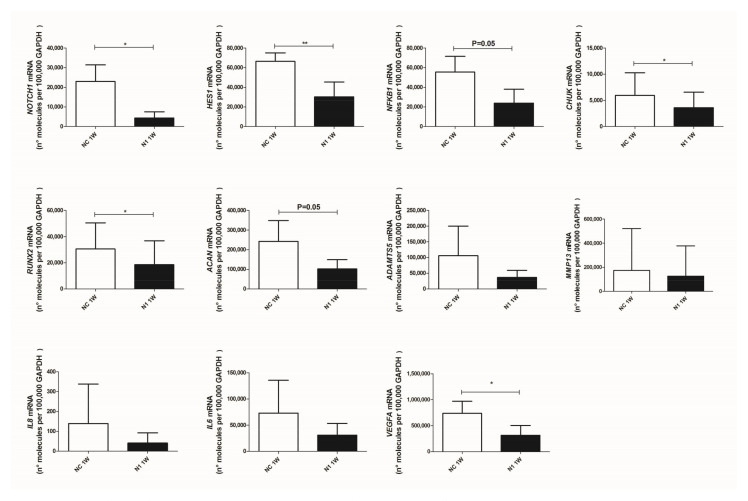
Effects of NOTCH1 silencing at the level of transcription of key genes in osteoarthritis. Total RNA was extracted from 1 week micromasses established with control (NC) or NOTCH1 silenced chondrocytes (N1). mRNA levels were calculated for each target gene and normalized using the reference housekeeping gene GAPDH according to the formula 2^-ΔCt^ and expressed as number of molecules per 100,000 GAPDH molecules; * *p* < 0.05, ** *p <* 0.01.

At first, we checked and confirmed the maintenance of NOTCH1 knockdown (Figure 4 upper left graph, showing that the level of knockdown remained high: 73 ± 30 (mean ± SD, *p* = 0.0134, *n* = 5).

Then, we assessed the effects on signaling intermediates and NOTCH1 targets. As expected, we found that HES1 was significantly reduced in NOTCH1 silenced micromasses. We also confirmed significantly reduced levels of CHUK (i.e., IKKα, one of the NF-κB upstream activating kinases and a NOTCH target gene [39]) and a reduction of NFKB1, i.e., NF-κB p105, one member of the family of NF-κB monomers recognized among the NF-κB target genes [40].

We then moved to assess expression of genes connected with progressed differentiation from resting to hypertrophic and terminal differentiated chondrocytes, in keeping with [3,4].

RUNX2, a pivotal transcription factor that drives hypertrophy [41] was significantly reduced by NOTCH1 silencing, as well as transcription of aggrecan, that continues across the chondrogenesis-hypertrophy maturation. Concerning the key matrix degrading enzymes in OA [42], a profound yet not significant reduction of ADAMTS5 was found in 3-D cultures with NOTCH1 silencing while milder effects were observed with MMP13, possibly because of a high degree of patient variability together with the effects of pre-activated NF-κB and p38 MAPK in high density cultures [43].

Lastly, we observed reduced levels of IL8 and IL6 and significant reduced (*p* = 0.018, *n* = 4) levels of VEGF, the master angiogenetic factor, responsible for neovascularization of late OA cartilage, thus reproducing the status of terminally differentiated cartilage of growth plate [4].

#### 2.4.2. Reduced Expression of RUNX2 Protein and Reduced Deposition of Glycosaminoglycan and Calcium 

**Figure 5:**
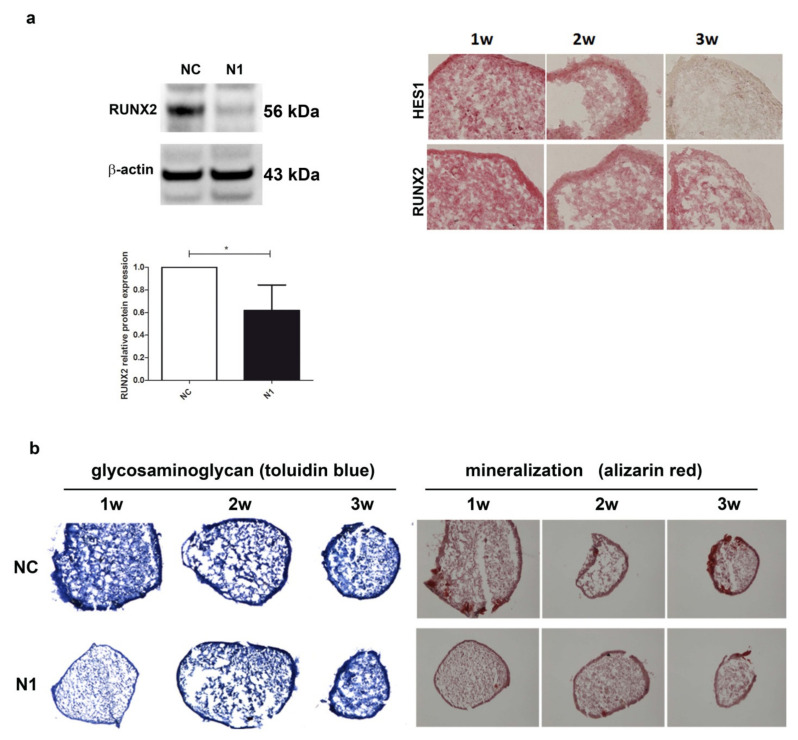
Effects of NOTCH1 silencing in the differentiation of chondrocytes in 3-D cultures. (**a**) NOTCH1 silencing impacts on RUNX2 expression: left, a representative Western blot indicates that RUNX2 protein is significantly reduced in NOTCH1 silenced 3-D cultures at 1 week as confirmed in the cumulative assessment shown in the lower graph (*n* = 5, * *p* < 0.05); right, immunohistochemistry imaging of the correlated reduced expression of RUNX2 and HES1 in micromass maturation across 1–3 weeks. (**b**) NOTCH1 silencing impacts on ECM maturation. Left, GAG deposition at 1, 2, and 3 weeks as evidenced by toluidin blue staining: GAG deposition resulted delayed in N1 KD 3-D constructs. Right: Alizarin Red staining on micromasses showed a markedly reduced calcium deposition at all time points in N1 KD micromasses. All images were obtained with a NIKON Eclipse 90i microscope equipped with 10× objective lens.

Given the importance of RUNX2 in the phenomena underlying OA progression [41], we further assessed the effect of NOTCH1 ablation on RUNX2 at the protein level (Figure 5).

The level of RUNX2 protein in 1 week old micromasses was significantly reduced, down to 60% of the level in control chondrocytes (*p* < 0.05, *n* = 5) as assessed by Western blot analysis with the normalization with reference to β-actin (Figure 5a). The reduced RUNX2 level paralleled that of HES1 in the 1w–3w maturation of N1 micromasses (Figure 5a right images). Consequently, the reduced RUNX2 determined a delayed maturation of the extracellular matrix, as shown by a markedly reduced glycosaminoglycan deposition at 1 week in N1 compared to NC micromasses. In parallel, alizarin red staining showed a markedly reduced deposition of calcium crystals, particularly evident at 1 week, but also maintained at 2 and 3 weeks. Supplementary Figure S8 shows the results of the image analysis of the pictures shown in Figure 5, performed with the Nikon Imaging Software (NIS).

#### 2.4.3. Reduced Release of Matrix Remodeling Enzymes

In keeping with the observed delayed maturation of 3-D chondrocyte cultures, we expected an effect on the repertoire of catabolic enzymes. The analysis was carried out in the supernatant of micromasses established with cells from four different patients. From each patient, triplicate micromasses were seeded and tested separately.

**Figure 6:**
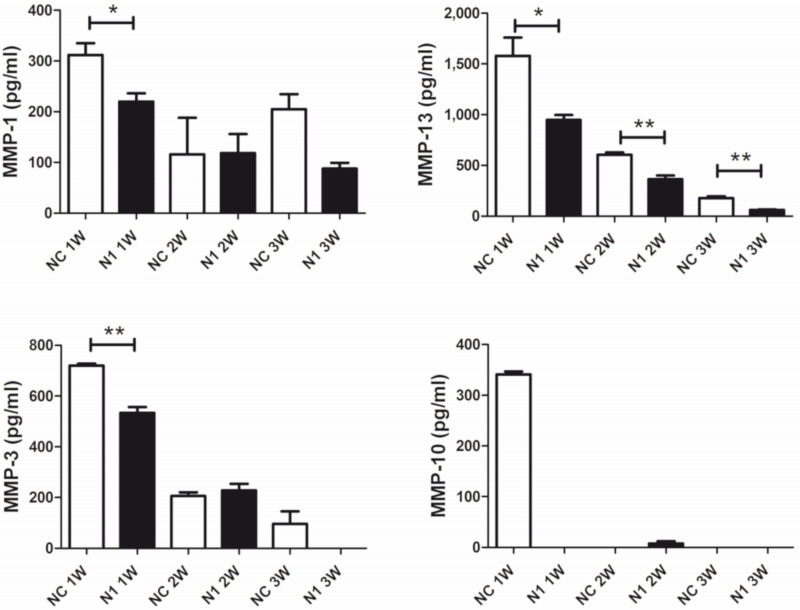
Effects of NOTCH1 silencing in the repertoire of MMPs released by chondrocytes cultured in 3-D. A representative patient out of four analyzed. Data are relative to triplicate micromasses at 1, 2, and 3 week maturation and are expressed as mean ± standard error of mean. Statistical analysis was performed by Student’s t test; * *p* < 0.05, ** *p <* 0.01.

The regulation of ECM remodeling downstream N1 KD and across differentiation (1–3 week maturation) of the 3-D cultures was investigated evaluating the released MMP repertoire by means of the Bio-Plex Pro™ Human MMP Panel, 9-Plex (Biorad). High levels of MMP-2 were found in the supernatant of micromasses, but unaffected by NOTCH1 silencing. On the other hand, levels of MMP-9, -8, -7, and -12 were below the calibration curve. Therefore, we focused on comparison of the levels of MMP-1, 3, 10, and 13 (Figure 6). In most cases, reduction of MMP release was evident at the beginning of maturation. Quantification of major collagenases MMP-1 and 13 and of stromelysin 1 (MMP-3) and 2 (MMP-10) showed statistically significant reduction after NOTCH1 KD. In particular, the reduction of MMP-10 in the supernatant of N1 KD samples at 1 week of maturation, suggested a decreased activation of collagenases.

#### 2.4.4. Reduced Terminal Differentiation and Increased Viability

**Figure 7:**
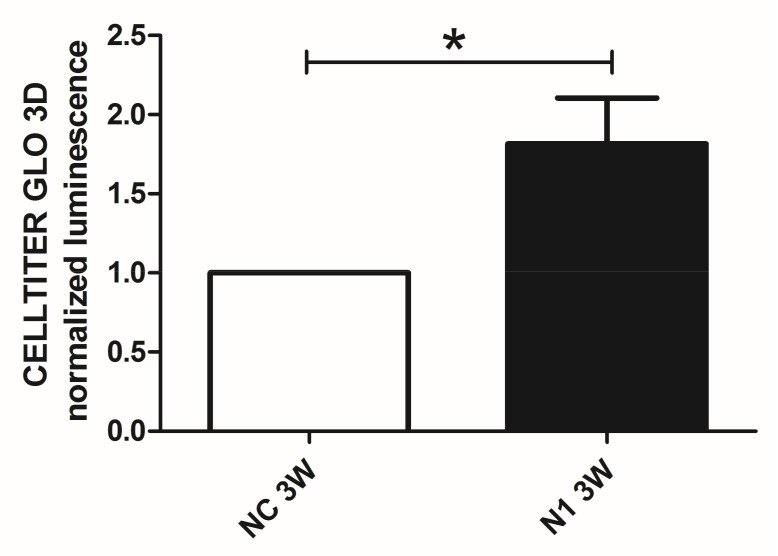
Effects of NOTCH1 silencing in the long term viability of chondrocytes cultured in 3-D. Assessment of cell viability in 3 week micromasses by means of the Cell Titer GLO 3D cell viability assay. N1 samples exhibited a statistically significant increased viability compared to NC. Data represent N1 luminescence normalized as fold change compared to NC values and are expressed as mean ± SD (*n* = 3). Statistical analysis was performed by Student’s t test for paired samples; * *p* < 0.05.

We previously showed that in 3 weeks’ time, chondrocytes cultured in 3-D recapitulate progression from hypertrophy to terminal differentiation with evidence of cell death that can be attenuated by means of selective targeting of critical effectors of progressed differentiation [29]. Therefore, to avoid biases connected to different viability we performed at 1 week maturation the assessment of the NOTCH1 dependent differential gene and protein expression of critical genes connected to hypertrophy. On the other hand, to maximize the effect of NOTCH1 silencing on terminal differentiation, we evaluated cell viability at 3 weeks as cell death is a hallmark of terminal differentiation (Figure 7). We found that NOTCH1 silencing significantly increases viability (*p* = 0.0396, *n* = 3) in chondrocytes cultured in 3-D compared to control chondrocytes, as evaluated by mean of the Cell Titer GLO 3D assay.

## 3. Discussion

Previous evidence of the increased NOTCH1 expression in OA cartilage in human and animal models has supported the hypothesis that NOTCH1 inhibition could be therapeutic in OA management, but recent evidence has suggested that this issue should be carefully managed [17], since complete abrogation of this pathway via NOTCH1 antisense in all joint tissues in the DMM model instead exacerbated experimental OA. In keeping with the activity of NOTCH being both context and time specific other authors have shown that while sustained NOTCH1 activation promotes OA changes, transient NOTCH activation is chondroprotective and enhances ECM synthesis [24]. The crucial role of NOTCH in both development and OA disease has also been recently reported in arthritis of the temporomandibular joint in rats [44].

The aim of our work was to assess the effects of NOTCH1 silencing 2-D and 3-D cultures established with primary human osteoarthritic chondrocytes. The silencing strategy was chosen to selectively target NOTCH1 and therefore both its canonical (i.e., RBPJ-dependent) and non-canonical effects while sparing the NOTCH2 pathway which is quite highly expressed in articular chondrocytes [15]. The latter would have been affected by other possible strategies to dampen NOTCH1 pathway such as the use of RBPJ siRNA. Collectively, our results confirm that NOTCH1 inhibition is effective in reducing the major hallmarks of chondrocyte phenotypic dysregulation in OA cartilage: proliferation and differentiation progression to hypertrophy and terminal differentiation driven by extracellular matrix remodeling. The latter processes, that must be prevented in healthy cartilage but that are improperly triggered in OA, are also driven by re-expression of NOTCH1 [6]. NOTCH1 overexpression induces hypertrophy in condrocytes in vitro and in vivo [45]. In contrast, our data show that NOTCH1 silencing delays differentiation in 3-D, based on reduced gene expression of differentiation markers, reduced calcium deposition, and reduced cell death. Therefore, NOTCH1 signaling inhibition may represent one of the constraints that prevent progression of the differentiation of articular chondrocytes toward hypertrophy. Indeed, across chondrogenesis and endochondral ossification, the expression of NOTCH1 is time and space restricted. In endochondral ossification, NOTCH1 is switched on at the stage of pre-hypertrophic and early hypertrophic chondrocytes [6].

Our findings on chondrocyte proliferation show biphasic effects of NOTCH1 inhibition, further confirming the complexity of the pathway. At early time points (48 h) NOTCH1 KD elicit higher proliferation based on a higher chondrocyte yield and higher fraction of cycling (S-G_2_M) cells. These findings are in keeping with those of Shang et al. [22], who showed that activation of the NOTCH1 signaling pathway via NICD1 transfection coordinately promoted cell cycle arrest (via Cyclin D1 inhibition and p57 induction) and chondrocyte hypertrophy (via SMAD1/5/8) [22]. However, findings at 48 h, based on cells collected after siRNA transfection are obtained before the completion of replication of primary chondrocyte cultures, since the estimated doubling time of chondrocyte cultures is 72 h [46,47]. On the other hand, at later times, NOTCH1 KD chondrocytes exhibited a lower proliferation, possibly because of a reduced expression of HES1, the main NOTCH1 target gene, known to be regulated by both NOTCH canonical and non-canonical pathways. Besides NOTCH, regulation of HES1 also involves other pathways [48], among which the Wnt-β-catenin and the Hedgehog pathways deserve to be mentioned for their role in chondrocyte differentiation. HES1 localizes in actively dividing cells confirming a role in controlling cell cycle progression, namely G_1_/S transition (Reactome pathway analyzer, https://reactome.org/, accessed on 25 March 2021), in keeping with [49]. Moreover, the ability of HES1 of reducing p27 [37] and p57 [50], two cyclin-dependent kinase inhibitors, has previously been reported. Our findings are also in agreement with those of Karlsson et al. who found reduced proliferation of normal human chondrocytes after NOTCH1 signaling antagonism with a γ-secretase inhibitor [51].

The requirement of high NOTCH1 levels in “proliferating” 2-D compared to the “differentiating” 3-D cultures has been confirmed by our Western blot results that show a much higher expression of NICD1 in the former. Moreover, the evidence of differential impact and function of the pathway in 2-D or 3-D cultures is in keeping with findings of Karlsson et al. [51] who assessed the regulation of NOTCH using normal human chondrocytes during proliferation or across differentiation recapitulated in micromass and found reduction of NOTCH related proteins in differentiating pellet cultures [51], mimicking the levels found in cartilage biopsies.

The connection between NOTCH1 expression and chondrocyte proliferation has also been reported by Khan and coworkers who treated cartilage explants with the mitogen FGF-2 [52] and found increased expression of NOTCH1 and related genes (MMP-13, ADAMTS, and HES-1). The same authors proved that the role of NOTCH1 in the context of cell proliferation was a non-canonical activity being insensitive to γ-secretase inhibitors.

With regard to the effects on chondrocyte differentiation recapitulated in 3-D micromass cultures, globally our results confirm that NOTCH1 signaling inhibition delays the progression towards hypertrophy, via reduced expression of RUNX2 and ECM remodeling. OA onset and progression are sustained by changes in ECM remodeling [41]. Increased ECM remodeling may derive from increased MMP expression along with increased activation and/or decreased inhibition by the TIMPs. To our knowledge, few studies have investigated the relationship of NOTCH signaling with ECM degrading enzymes in articular chondrocytes, and mainly using murine chondrocytes. Indeed, in the latter settings, Sugita et al. described HES1 responsive elements in ADAMTS-5 and MMP-13 genes [23]. RBPJ, the primary nuclear mediator of NOTCH canonical activity upregulates RUNX2 promoter transcriptional activity [53]. NOTCH signaling in chondrocytes has been previously linked to increased MMP expression, particularly of MMP-13 that represents the pivotal collagenase in cartilage degradation [41]. A first study suggested a direct connection between MMP-13 and NOTCH: employing NOTCH signaling inhibition (DAPT) on murine chondrocytes a significant reduction of MMP-13 mRNA and protein was found [25], particularly in cells at later passages while MMP-2 resulted unaffected (as also found in supernatants from NC and N1 micromasses). A similar study was reproduced by Sassi et al. using human healthy articular chondrocytes from young donors. These cells do not express MMP-13 at early passages, while MMP-13 expression is induced by serial subculturing (at p3), together with that of Col1 and eNOS (dedifferentiation markers) preceded by that of NOTCH1, found at p2. At the same time, expression of differentiation markers of “healthy chondrocytes” such as collagen 2 and aggrecan decreased, while the addition of DAPT was able to restore collagen 2 expression at p3 [27].

Blaise and Sassi therefore suggested that NOTCH signaling was pivotal in “chondrocyte dedifferentiation”, but findings collected when the chondrocyte phenotype is lost, such as at the third passage, are less informative compared to those collected with primary chondrocytes cultured in 3-D [38,54], a culture model that allows for the recovery of the correct phenotype of the chondrocytes, in close relationship with their ECM [55,56]. Some differential cell reaction patterns of phenotypic modulation have been previously described in OA chondrocytes [57], but the current view is that rather than to “dedifferentiation”, changes of chondrocyte features in OA are better related to a loss of maturational arrest, i.e., to a “progressed differentiation” into endochondral ossification and toward terminal differentiation [3,58], a process that can be reproduced dynamically in vitro in 3-D (micromass) cultures. Indeed, using this model and primary OA chondrocytes, we were previously able to show the effects of targeting critical effectors or signaling intermediates [29,59,60] of this process. The status of chondrocytes within 3-D cultures is similar to that of chondrocytes in cartilage explants but is more amenable for a large and multiplexed series of analyses [61].

The chondroprotective and anabolic effects of DAPT-dependent NOTCH1 inhibition was also confirmed in human cartilage: OA cartilage explants expressed MMP-13 but this expression was abrogated upon DAPT treatment, that was also able to rescue collagen II and aggrecan expression [27]. In the context of NOTCH1 pathway inhibition, it should be underlined that our data obtained with siRNA mediated NOTCH1 silencing are more selective compared to those obtained with DAPT, that at least also inhibits the other NOTCH receptors.

Other studies exploiting transfection of NICD in murine chondrocytes have shown that NOTCH1 induces MMP-13 via IL6-mediation [62]. Moreover, NOTCH signaling is involved in the expression of many other genes relevant in OA pathophysiology, such as IL-8 and MMP-9 [16].

The NOTCH-MMP-13 connection was confirmed with several other findings derived from both in vivo and in vitro studies. In a mouse model with selective inhibition of NOTCH signaling (via SOX-9-Cre; Rbpj fl/fl), a significant decrease was observed of markers related to endochondral ossification (MMP-13) and angiogenesis (VEGF-A), that paralleled the decrease of HES1, the master target gene of NOTCH1 [15]. A similar pattern of regulation was observed in an inducible and articular cartilage specific murine model (Col2a1-Cre; Rbpj fl/fl) where inactivation of Rbpj signaling was executed at skeletal maturity: this mouse was resistant to OA development as induced by means of surgical induction of joint instability, and this resistance was phenocopied with intraarticular delivery of DAPT [15]. More recently, it was shown that inducible and articular specific HES1 ablation in mice (in a Col2a1-CreERT; Rbpj fl/fl background) prevented OA progression, and inhibited expression of the key matrix degrading enzymes in OA, i.e., MMP-13 and ADAMTS-5 [23].

The NOTCH1 connection with chondrocyte hypertrophy and MMP-13 expression was shown in a recent study that used the murine chondrocytic cell line ATDC5. In these settings, transfection of NICD1 resulted in significant Sox9 reduction and Runx2 induction (and of alkaline phosphatase), suggesting that NOTCH1 signaling has profound effects on chondrocyte differentiation [22].

In agreement with our findings, a recent report of both in vitro and in vivo (rats) experiments has further addressed the relationship between NOTCH1, RUNX2, and MMP-13 [26] by either activating (with Jagged-1, the relevant ligand) or inhibiting (by DAPT treatment) the NOTCH1 pathway in conjunction with strategies of RUNX2 modulation via either silencing shRNA or overexpressing plasmids.

In our 3-D model, we observed reduced expression of RUNX2 and MMP-13 following NOTCH1 RNA interference. Moreover, our bioplex assay showed significantly reduced expression of MMP-1, the other collagenase relevant in OA [63], in NOTCH1 KD cells. In addition, we observed markedly reduced expression of MMP-10, an enzyme that contributes to activation of collagenases in OA [64], besides decreased expression of MMP-3, a stromelysin included among the serum biomarkers of OA activity [65].

Some recent reports have also indicated the occurrence of relevant crosstalks in cartilage between the NOTCH1 and the NF-κB pathways [8,9], the latter exerting a fundamental role in OA onset and progression [66]. In addition, in 1 week micromasses established with NOTCH1 KD chondrocytes, we observed reduced gene expression of IKKα, a gene that we highlighted as having a role in OA pathophysiology [29,60,67,68] and that has also been included among the known NOTCH targets [39], although not much information is available with regard to human chondrocytes. IKKα might play multiple roles in OA pathophysiology. It contributes to NF-κB canonical activation [69], it is pivotal in NF-κB delayed non canonical activation, and also plays a peculiar role in chromatin remodeling required for NF-κB transcription [70,71]. In breast cancer cells, IKKα favors the chromatin recruitment of NOTCH1 transcriptional complex (NTC) and is recruited to the NTC itself in a NOTCH dependent manner [72]. Moreover, NOTCH1–IKKα interaction has been shown to exert an anti-apoptotic role [73]. In a rheumatoid arthritis mouse model, the IKKα-dependent non-canonical heterodimer relB-p52 enhances the transcriptional activity of NOTCH and Rbpj, required for the transcription of ADAMTS5 and MMP-13 [8]. In the latter disease context recent evidence has shown the feasibility of nanomedicine approaches for the intra-articular delivery of NOTCH1 siRNA-coupled micelles in a rheumatoid arthritis model developed in rats [74]. This approach is amenable to fine tuning in order to spare the chondroprotective and anabolic activities of the NOTCH1 pathway.

## 4. Materials and Methods

### 4.1. Establishment of Chondrocyte Cultures

Primary cultures of human osteoarthritic chondrocytes were established from knee cartilage derived from total knee replacement in OA patients. The study was conducted according to the guidelines of the Declaration of Helsinki and approved by the Ethics Committee of Istituto Ortopedico Rizzoli (ethic approval code: 0019715, approved on 28 September 2016), including documentation of written patient informed consent. 

The patients (5 females and 1 male) had a mean ± SD age of 74 ± 2. The patients admitted to the study had to meet inclusion (patients undergoing total knee replacement, aged more than 18, and able to autonomously express the informed consent to participate to the study) and exclusion criteria (the latter criteria excluded patients with BMI higher than 35, or with complicating disease such as rheumatic diseases, diabetes, severe chronic infective diseases or malignancies, severe psychiatric diseases or use of steroid drugs or insulin). These patients had Kellgren-Lawrence (KL) grade 3 or 4.

After tissue retrieval, all patient identifiers were removed, and samples were coded by arbitrary designations to distinguish them solely for experimental purposes. Primary chondrocytes were isolated by mean of sequential enzymatic digestion (1 h with pronase (Sigma-Aldrich, Merck KGaA, Darmstadt, Germany)) and 1–2 h with 0.2% collagenase (Sigma-Aldrich) at 37 °C) from cartilage (*n* = 6, age = 73.8 ± 1.72, mean ± SD), as described in [75]. After recovery, isolated chondrocytes were filtered by 100 and 70 μm nylon meshes, washed, centrifuged, and counted. Chondrocytes were cultured in 10% FCS D-MEM (Sigma-Aldrich) with the addition of antibiotics in T150 flasks at an initial seeding density of 20.000 cells for cm^2^ and left to grow until confluence. Only P_0_ chondrocytes (i.e., cells that did not undergo subculturing and therefore retaining proper chondrocyte differentiation) were used for silencing experiments. 

Some experiments were also performed with C28/I2 cells, an immortalized cell line widely used to mimic the behavior of primary chondrocytes [76].

### 4.2. Small Interfering RNA Mediated NOTCH1 Gene Silencing 

NOTCH1 silencing of several chondrocyte cultures was obtained by mean of RNA interference (RNAi), using ON-TARGETplus SMARTpool with si-NOTCH1 or ON-TARGET plus Non-targeting Pool, Dharmacon (Horizon Discovery, Perkin Elmer, Cambridge, UK). The SMARTpool is a mixture of four siRNA providing advantages in both potency and specificity. Transient transfection was performed by Lipofectamine RNAiMAX Transfection Reagent (ThermoFisher Scientific, Waltham, MA, USA). Then, 48 h after siRNA delivery, chondrocytes were collected for count and evaluation of NOTCH1 knockdown (KD) at both gene and protein level, and for either monolayer or micromass seeding. Some cells were fixed in ethanol for subsequent cell cycle analysis. Given the high variability at the level of gene expression in the different primary cultures evaluated, the comparison was performed after variance normalization by using the Log_10_ of the values.

### 4.3. Cell Cycle Assessment 

Flow cytometry was employed to evaluate cell cycle by means of DNA staining (Sytox green at 5 μM, Molecular Probes, ThermoFisher Scientific) of cells previously fixed with 70% ethanol and RNAse treated: 2.5 U RNAse One (Promega, Madison, WI, USA) plus 100 μg/mL RNAse A, Sigma-Aldrich). Analyses were performed using a FACS Canto II flow cytometer (Becton Dickinson Biosciences, Franklin Lakes, NJ, USA).

### 4.4. Chondrocyte Cultures 

The 2-D culture was employed to evaluate the effect of NOTCH1 silencing on chondrocyte growth by means of the Picogreen assay as described below performed in combined NC and N1 cultures collected at 48 h after transfection. 

The 3-D cultures established as described in [29] were allowed to mature across 3 weeks, with medium changes every second day to assess the effects of NOTCH1 silencing on differentiation progression. At selected time points (1w, 2w, and 3w) parallel samples were either embedded in OCT compound for immunohistochemistry and alizarin red or toluidine blue staining as described in [59] or dry frozen for subsequent Western blot or real time RT-PCR analysis or viability assay. Supernatants were also collected for MMP measurement. Noteworthy, for each experimental condition at least 4–6 replicate were established, and MMP assessment was carried out in triplicate for each of the four cultures assessed.

### 4.5. PicoGreen Assessment of Cell Growth

The analysis of cell growth was undertaken essentially as reported in [29], by means of a quantitative (Quant-IT PicoGreen dsDNA assay kit, Molecular Probes, ThermoFisher Scientific) DNA analysis of the proliferating cells. The fluorescence signal was collected from the bottom of the wells exploiting the well scan mode (3 × 3 areas) of the Spectra Max Gemini plate fluorometer (Molecular Devices, Sunnyvale, CA, USA).

NOTCH1 KD and control chondrocytes were seeded at low density (1000 cells per well in quintuplicate) in 96 well plates 48 h after transfection and cultured for 12 days. Parallel plates were established in order to measure the cell growth at times 0 (the day after seeding), 3, 7, 10, and 12, using one plate for each chondrocyte phenotype (NC or NOTCH1 KD) for each time point. At the selected time points, the plates were emptied and frozen at −20 °C until analysis, that was performed at the same time for cultures established from the same patient. To correct for differences in cell counts, values were calculated as the percentage increase over the starting (day 0) values [29]. Images of the wells were also obtained by using an inverted Nikon (Nikon Corporation, Tokio, Japan) Eclipse TS100 microscope equipped with a 465-495EX, 505DM, 515-555BA nm filter to collect the green signal of the stained nuclei. 

### 4.6. Western Blot

Western blot was carried out to assess the differential NOTCH1 level in 2-D and 3-D cultures and to assess the level of KD efficiency, after siRNA delivery. The experimental details were essentially as described in [75]. Proteins from equal cell equivalents (150,000 cells) or micromasses (one half 1 w micromass per well, established with 250,000 cells) were extracted by means of Lysis buffer supplemented with benzonase to improve extraction of proteins bound to DNA, besides NaF, Na_3_VO_4_, PMSF, and Protease Inhibitor cocktail. In the case of 3-D cultures, improved extraction was achieved by using pestles connected to a pellet pestles cordless motor (Sigma-Aldrich). Extraction was carried out on ice (30 min), with 10 s vortexing every 10 min. At the end, the samples were centrifuged for 15 min at 10,000 rpm at 4 °C. The supernatants were collected and kept at −80 °C until analysis. Lysates corresponding to 150,000 cells (in 10 µL) or half micromasses (in 15 µL) were loaded in wells of NuPage pre-cast Bis-Tris 4–12% (ThermoFisher Scientific), after addition of LDS NuPage sample buffer and NuPage reducing agent and boiling to 100 °C for 10 min then transferred to ice. Electrophoretic run of proteins exploited MOPS or MES as a running buffer. In each gel, a molecular weight marker (Novex Sharp pre-stained protein standards, ThermoFisher Scientific) was loaded along the samples to allow accurate estimation of correct molecular weight. At the end of the run, the proteins were transferred to PVDF (Immobilon-P, Millipore, Merck) membranes exploiting the iBlot Dry Blotting system (ThermoFisher Scientific). To allow retention of proteins, the membranes were dried by immersing them in methanol, and stored at 4°C until use. At the time of analysis, the membranes were rehydrated by methanol and subjected to Western blot by means of the SNAP-ID 2.0 device (Millipore, Merck) or by conventional overnight incubation. Primary antibodies were as follows: NOTCH1 (polyclonal rabbit anti-human NOTCH1, sc-6014R used at 0.1 µg/mL, Santa Cruz Biotechnology, Dallas, TX, USA), cleaved Notch1 (Val1744) (rabbit monoclonal, #4147 used 1:1000, Cell Signaling Technology, Danvers, MA, USA), RUNX2 (polyclonal goat anti-human RUNX2, AF2006 used at 1:20,000, R&D Systems, Minneapolis, MN, USA). β-actin (mouse monoclonal, #A2228, clone AC-74, used at 0.8 µg/mL, Sigma-Aldrich) or GAPDH (mouse monoclonal, MAB374, used at 0.8 µg/mL, Sigma-Aldrich) were used as loading controls. Appropriate HRP conjugated anti secondary antibodies anti species (mouse, rabbit, goat) immunoglobulins were from Jackson ImmunoResearch Europe (Cambridge, UK) and the substrate was ECL-Select (Amersham, Cytiva, Marlborough, MA, USA). Signals were acquired by means of a ChemiDoc MP Imaging System (Bio-Rad Laboratories, Hercules, CA, USA). For accurate assessment of molecular weight, the signals were referred to the molecular weight marker stained with a chemiluminescent pen (Glow Writer, Sigma-Aldrich). With regard to NOTCH1, Western blot experiments showed occurrence of multiple bands, in keeping with the existence of intracellular enzymes in charge of cleavage and/or ubiquitination [77]. Therefore, for quantitative purposes we considered the intensity of the band corresponding to the NICD1 fragment, that in our hands resulted in 105-110 kDa. Semi-quantitative analysis of band intensity was performed considering “optical density” values and using Image Lab software (version 6.0, Bio-Rad). Samples were compared considering the expression level of the band of interest normalized to that of the housekeeping control.

### 4.7. Immunofluorescence and Immunohistochemistry

Immunofluorescence was carried out essentially as in [67] to disclose the pattern of NOTCH1 and HES1 in chondrocytes cultured on chamber slides, in relationship to mitosis or interphase.

For this purpose, NC and N1 chondrocytes at 72 h post-transfection were seeded onto chamber slides (8 well chamber slides, at a density of 10,000 cells per cm^2^) and let to adhere for 72 h. Then, after a brief washing with PBS the cells were fixed with 100 µl of 4% paraformaldehyde (PFA) for 30 min at RT and washed again with PBS. The wells were filled with PBS and stored at 4 °C until the time of processing. The samples were pretreated for antigen unmasking with 0.02 U/mL Chondroitinase ABC (Sigma-Aldrich) in 50 mM pH 8.0 Tris/HCl solution for 20′ at 37 °C and permeabilized with 0.2% Triton in TBS (TRIS buffered saline) solution for 5 min at RT. After another wash with TBS, the nonspecific bindings were blocked with a 5% BSA (bovine serum albumin, Sigma-Aldrich), 5% Normal Donkey serum (Jackson ImmunoResearch Europe), and 0.1% Triton in TBS for 30′ at RT and washed again. Then, primary and control antibodies were delivered at a concentration of 5 µg/mL in TBS with 3% BSA 0.1% Tween and left overnight at 4 °C: anti NOTCH1 (rabbit anti NOTCH1 (NBP1-78292, Novus Biologicals, Centennial, CO, USA), anti-HES1 (rabbit anti-HES1, PA5-28802, ThermoFisher Scientific), or control antibody (normal rabbit immunoglobulins, AB105-C, R&D Systems). After rinsing in TBS, the signal was revealed by a 15 µg/mL donkey anti-rabbit Alexa Fluor 555 secondary antibody conjugate (ThermoFisher Scientific) in TBS with 3% BSA 0.1% Tween and incubated 30′ at RT together with 1µg/mL Hoechst 33342 (Sigma- Aldrich) for nuclear counterstaining. At the end, the samples were mounted with the addition of anti-fading (1% 1,4 Diazobicyclo (2.2.2) Octane (DABCO, Sigma-Aldrich) in 90% glycerol, in 0.1 M pH 8.0Tris-HCl), sealed with nail-polish and stored refrigerated and in the dark for subsequent analysis.

Images of these experiments were observed by mean of a NIKON (Nikon Corporation) Eclipse 90i microscope equipped with 540/25EX, 565DM, 605/55BA nm filters to evaluate NOTCH1 or HES1 stained with a red emitting fluorochrome while nuclear counterstaining with Hoechst 33342 was evaluated with 330-380EX, 400DM, 420BA nm filters.

Immunohistochemistry was instead carried out to assess the correlated expression levels of RUNX2 and HES1 along the maturation of N1 micromasses. To this end, 5 µm sections were obtained from micromasses established with NC or N1 chondrocytes and embedded in OCT compound. The slides were put on silanized glass slides and stored at −20 °C. At the time of processing, the slides were left to equilibrate to room temperature still wrapped with aluminum foil. The areas bearing the micromass slide for immunodetection were delimited with the pap pen and fixed with 4%PFA for 30 min at RT, followed by washing. Here again, a step of antigen unmasking was carried out with 0.02 U/mL Chondroitinase ABC (Sigma-Aldrich) followed by blocking of nonspecific bindings as detailed above. Then, primary antibodies and control antibodies were delivered at a concentration of 5µg/mL in TBS with 3% normal goat serum, 2% BSA, and 0.1% Triton and left 2 h at RT: anti RUNX2 (rat monoclonal MAB2006 used at 5 µg/mL, R&D Systems) and anti-HES1 (rabbit polyclonal PA5-28802 used at 5 µg/mL, ThermoFisher Scientific) or control rabbit antibody (normal rabbit immunoglobulins BD AB105-C, R&D Systems). In addition, a sample with only secondary antibody was set up, to control for nonspecific binding of immunodetection reagents. At the end of the incubation with primary antibodies two washings with TBS were performed, and immunodetection was carried out using the supersensitive IHC Detection System (BioGenex, Fremont, CA, USA) suitable to detect mouse, rat, or rabbit primary antibodies exploiting the avidin-biotin amplification system, to localize the alkaline phosphatase enzymes at antigen sites finally revealed with FAST RED substrate. At the end, the sections were washed and mounted with Aquamount. Pictures at 200× magnification (objective 20×) were obtained with Eclipse 90i Nikon.

To provide quantitative assessment of signal intensity, results of immunohistochemistry, toluidin blue, and alizarin red staining were analyzed with the Nikon Imaging Software (NIS, Nikon Corporation). For each signal, tissue areas underwent a thresholding using the intensity function of the software. NIS identified several areas (from tens to hundreds for each section with more areas in sections with higher staining) and produced an output with several calculated parameters, including mean intensity and area for each. These data were used in a statistical analysis (ANOVA) to compare the conditions. To emphasize the information that the cumulative area identified by NIS as “beyond the threshold” was variable according to the specific condition, we also included the graphical representation of the product of the mean intensity and the cumulative area.

### 4.8. MMPs Quantitative Assessment 

Supernatants of micromasses derived from control siRNA (NC) or NOTCH1 siRNA (N1) chondrocytes were used to test the role of NOTCH1 on the expression of major matrix metalloproteinases, across micromass maturation.

To this end we used the Bio-Plex Pro™ Human MMP Panel, 9-Plex (Bio-Rad Laboratories, #171AM001M), a multiplex bead based sandwich immunoassay kit, with high sensitivity and dynamic range, to simultaneously evaluate the three collagenases (collagenase1/MMP-1, collagenase 2/MMP-8 and collagenase 3/MMP-13), the two stromelysins (stromelysin1/MMP-3 and stromelysin2/2MMP-10), the two gelatinase A (72 kDa, MMP-2) and B (92 kDa, MMP-9), the matrylisin/MMP-7, and the macrophage metalloelastase MMP-12.

### 4.9. Evaluation of Progressed Chondrocyte Terminal Differentiation/Cell Viability

Loss of maturational arrest leads to chondrocyte hypertrophy and terminal differentiation. This process can be recapitulated in vitro since 3w micromasses present a considerable number of dead cells that are reduced by selective targeting of effectors of chondrocyte hypertrophy [29]. Therefore, to test the effects of NOTCH1 silencing we used a recently developed assays: the CellTiter-Glo 3D Cell viability assay (Promega), that relies on the properties of a proprietary thermostable luciferase (Ultra-Glo™ Recombinant Luciferase), which generates a stable “glow-type” luminescent signal that is proportional to the amount of ATP present. The homogeneous “add-mix-measure” format results in cell lysis, “extraction” of intracellular ATP from healthy and viable cells, generation of a proportional luminescent signal, and good performance across a wide range of assay conditions.

Luminescence readings were collected from micromasses left to mature for 3 weeks and readings of NOTCH1 siRNA samples were normalized to their relative CTRLsiRNA samples. NOTCH1siRNA increased viability of 3 weeks samples of 1.82 ± 2.29 (mean ± standard deviation, *p* = 0.04, *n* = 3) fold. 

### 4.10. Real-Time RT-PCR Analysis

Total RNA was extracted from cell pellets collected at 48 h from NOTCH1 silencing and micromasses (1w maturation), with TRIzol RNA isolating agent (ThermoFisher Scientific) according to manufacturer’s instructions. Total RNA (1 µg) was reverse-transcribed using SuperScript VILO cDNA synthesis kit (ThermoFisher Scientific) following manufacturer’s protocol. Real-time RT PCR analysis were performed employing QuantiTect SYBR Green PCR Kit (TaKaRa Bio inc., Shiga, Japan) and following standard protocol: Taqman DNA Polymerase activation 95° (45 cycles; denaturation 95°, amplification annealing temperature variable according to primers design, as indicated in Table 1). Results of mRNA quantification for both N1 and NC samples analysis were mRNA levels calculated for each target gene and normalized using the reference gene GAPDH according to the formula 2-ΔCt and expressed as a percentage of the reference gene. Primer specificity was established by evaluation of melting curves. Target gene primers sequences are shown below (Table 1).

### 4.11. Statistics

Data are represented as mean ± standard deviation (SD) and compared by means of two tailed Student’s t test using the GraphPad Prism 5.0 software (GRAPHPAD SOFTWARE, La Jolla, CA, USA). Differences were considered significant when *p* < 0.05 with * *p* < 0.05; ** *p* < 0.01; *** *p* < 0.001. 

## Figures and Tables

**Table 1:** List of primers used for real time RT-PCR.

*Gene*	*Forward Primer*	*Reverse Primer*	*Amplicon Size*
			*(Annealing T)*
*GAPDH*	TGGTATCGTGGAAGGACTCA	GCAGGGATGAGTTCTGGA	*123 bp (56 °C)*
*NOTCH1*	CCTGAAGAACGGGGCTAACA	GATGTCCCGGTTGGCAAAGT	*127 bp (60 °C)*
*HES1*	AAGAAAGATAGCTCGCGGCA	TACTTCCCCAGCACACTTGG	*134 bp (60 °C)*
*ADAMTS5*	GCACTTCAGCCACCATCAC	AGGCGAGCACAGACATCC	*187 bp (58 °C)*
*MMP-13*	TCACGATGGCATTGCT	GCCGGTGTAGGTGTAGA	*277 bp (58 °C)*
*ACAN*	TCGAGGACAGCGAGGCC	TCGAGGGTGTAGCGTGTAGAGA	*85 bp (60 °C)*
*RUNX2*	GGAATGCCTCTGCTGTTATG	AGACGGTTATGGTCAAGGTG	*105 bp (58 °C)*
*NFKB1*	CAGGAGACGTGAAGATGCTG	AGTTGAGAATGAAGGTGGATGA	*109 bp (60 °C)*
*CHUK (IKKα)*	GCACAGAGATGGTGAAAATCATTG	CAACTTGCTCAAATGACCAAACAG	*86 bp (60 °C)*
*IL6*	TAGTGAGGAACAAGCCAGAG	GCGCAGAATGAGATGAGTTG	*184 bp (60 °C)*
*IL8*	CCAAACCTTTCCACCC	ACTTCTCCACAACCCT	*153 bp (60 °C)*
*VEGFA*	TGATGATTCTGCCCTCCTC	GCCTTGCCTTGCTGCTC	*82 bp (58 °C)*

## Data Availability

Data reported in the study are available upon request to the corresponding author.

## Supplementary Materials

The following are available online at https://www.mdpi.com/article/10.3390/ijms222112012/s1, Figure S1: full blots used to derive the NICD1 and GAPDH results shown in Figure 1a of the main manuscript; Figure S2: full blots used to derive the NICD1 and β-actin results shown in Figure 1b of the main manuscript; Figure S3: full blots used to derive the NOTCH1/NICD1 and β-actin results useful to compare NOTCH1/NICD1 intensity in both C28/I2 immortalized chondrocytes and primary chondrocytes cultured at either low density (LD) or high density (HD); Figure S4: full blots used to compare the signal of Cleaved NOTCH1 (Val1744) in C28/I2 immortalized chondrocytes or primary chondrocytes in basal conditions or after 1 hour stimulation with either 5 mM EDTA or 2.5 ng/ml IL-1β; Figure S5: full blots used to derive the NICD1 and GAPDH results shown in Figure 2a of the main manuscript obtained with lysates of chondrocytes in either control (NC, control siRNA) or NOTCH1 KD conditions; Figure S6: full blots (used to derive the NICD1 and β-actin results) and real time PCR results useful to assess the persistence of NOTCH1 silencing across 1, 2 and 3 weeks’ maturation of micromasses; Figure S7: full blots used to derive the RUNX-2 and β-actin results shown in Figure 5 of the main manuscript, obtained with lysates of chondrocytes cultured in 3-D, in either control (NC, control siRNA) or NOTCH1 KD (N1, NOTCH1 siRNA) conditions; Figure S8: image analysis of the immunohistochemistry (HES1 and RUNX2), toluidin blue and alizarin red results shown in Figure 5.
