# Supplementary material for: Pleiotropic Roles of NOTCH1 Signaling in the Loss of Maturational Arrest of Human Osteoarthritic Chondrocytes
Source: Int J Mol Sci. 2021 Nov 5;22(21):12012. doi: 10.3390/ijms222112012 (PMC8585104; doi:10.3390/ijms222112012)
Supplement: Supplementary file 1 [file ijms-22-12012-s001.zip › ijms-1320137-supplementary.pdf]

SUPPLEMENTARY MATERIAL

Pleiotropic roles of NOTCH1 signaling in the loss of maturational arrest of human osteoarthritic chondrocytes

Manuela Minguzzi[1†], Veronica Panichi[2†] , Stefania D'Adamo[1],

Silvia Cetrullo[2], Luca Cattini[3] , Flavio Flamigni[2], Erminia Mariani[1,3]

and Rosa Maria Borzì [3,*]

1 Dipartimento di Scienze Mediche e Chirurgiche, Università di Bologna, 40138 Bologna, Italy; manuela.minguzzi@gmail.com (M.M.); stefania.dadamo2@unibo.it (S.D.); erminia.mariani@ior.it (E.M.)
2 Dipartimento di Scienze Biomediche e Neuromotorie, Università di Bologna, 40138 Bologna, Italy; veronica.panichi2@unibo.it (V.P.); silvia.cetrullo@unibo.it (S.C.); flavio.flamigni@unibo.it (F.F.)
3 Laboratorio di Immunoreumatologia e Rigenerazione Tissutale, IRCCS Istituto Ortopedico Rizzoli, 40136 Bologna, Italy; rosamaria.borzi@ior.it (R.M.B.); luca.cattini@ior.it (L.C.)
* Correspondence: rosamaria.borzi@ior.it
† These authors contributed equally to this work.

S1

a

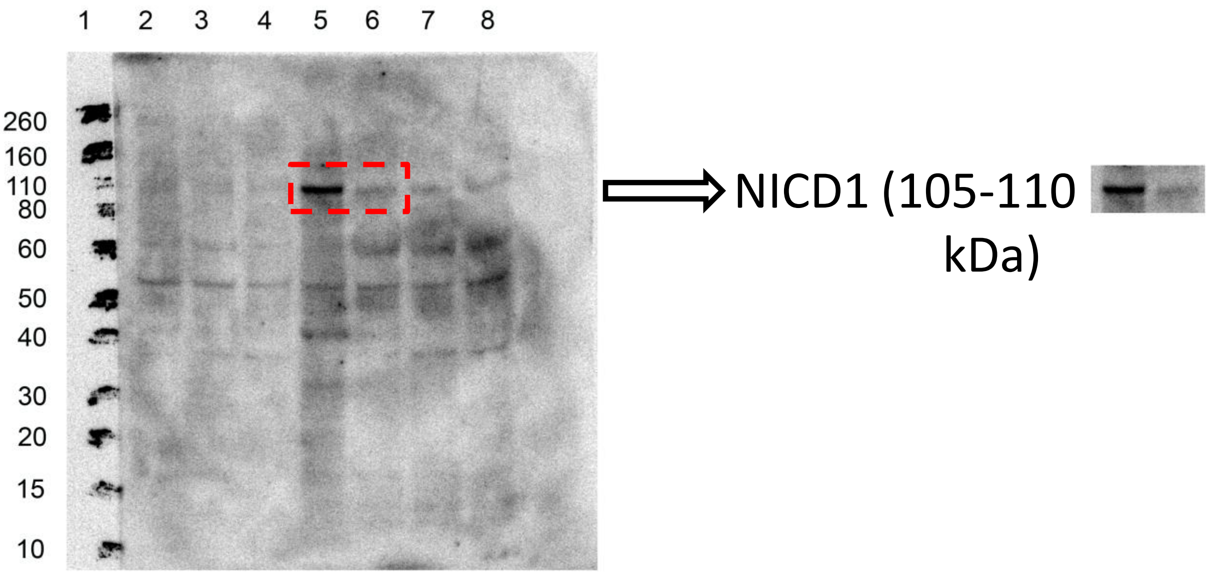

1 2 3 4 5 6 7 8
260
160
110
80
60
50
40
30
20
15
10
NICD1 (105-110 kDa)

b

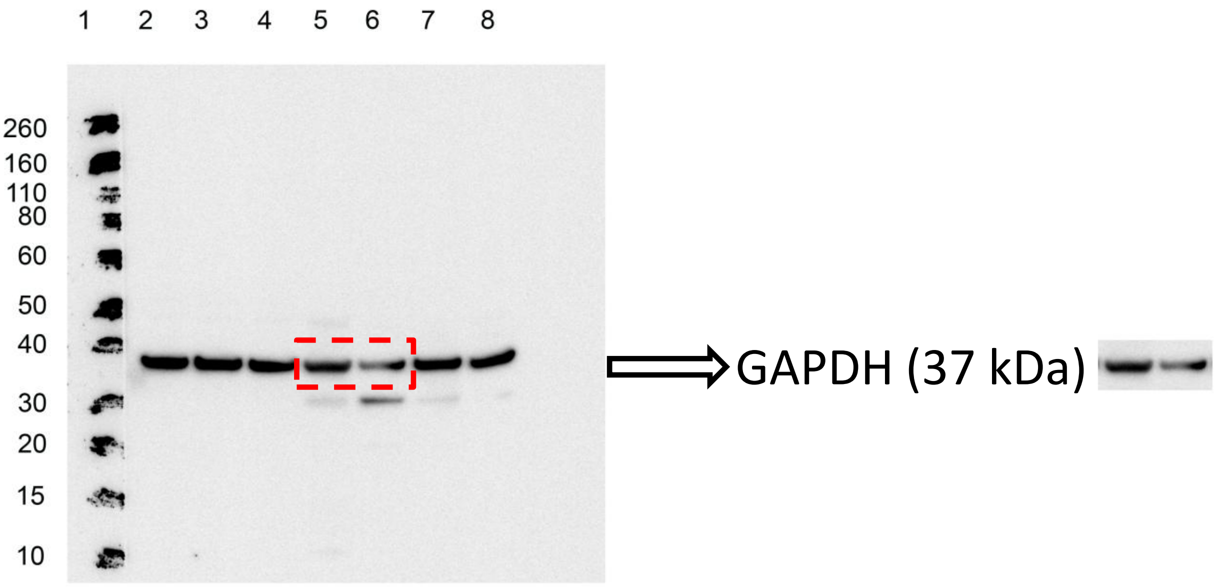

1 2 3 4 5 6 7 8
260
160
110
80
60
50
40
30
20
15
10
GAPDH (37 kDa)

S2

a

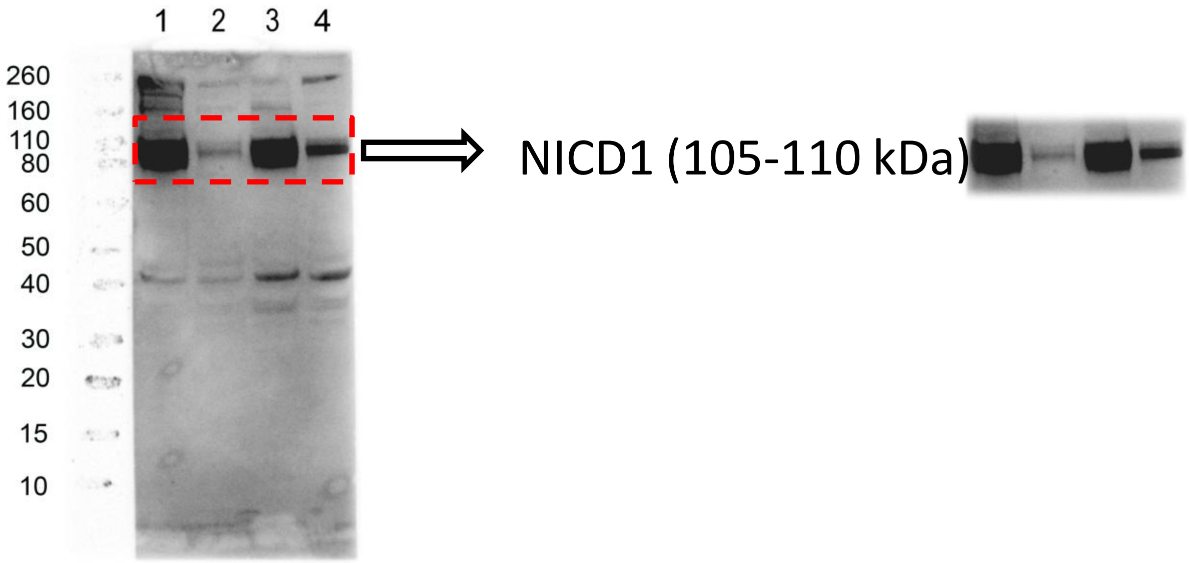
1 2 3 4
260
160
110
80
60
50
40
30
20
15
10
NICD1 (105-110 kDa)

b

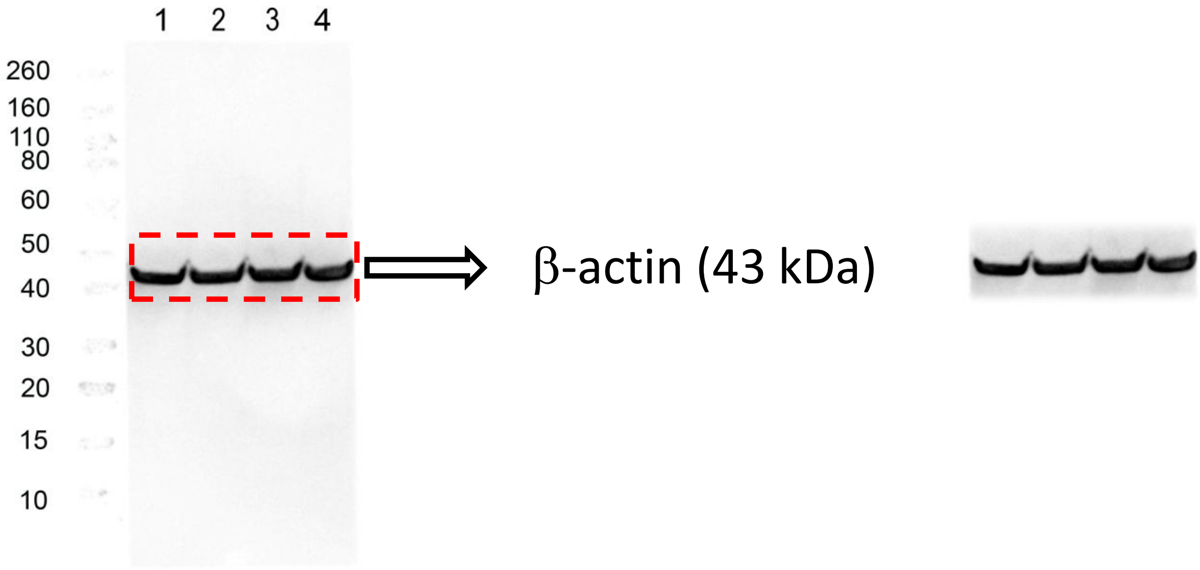
1 2 3 4
260
160
110
80
60
50
40
30
20
15
10
β-actin (43 kDa)

S3

a

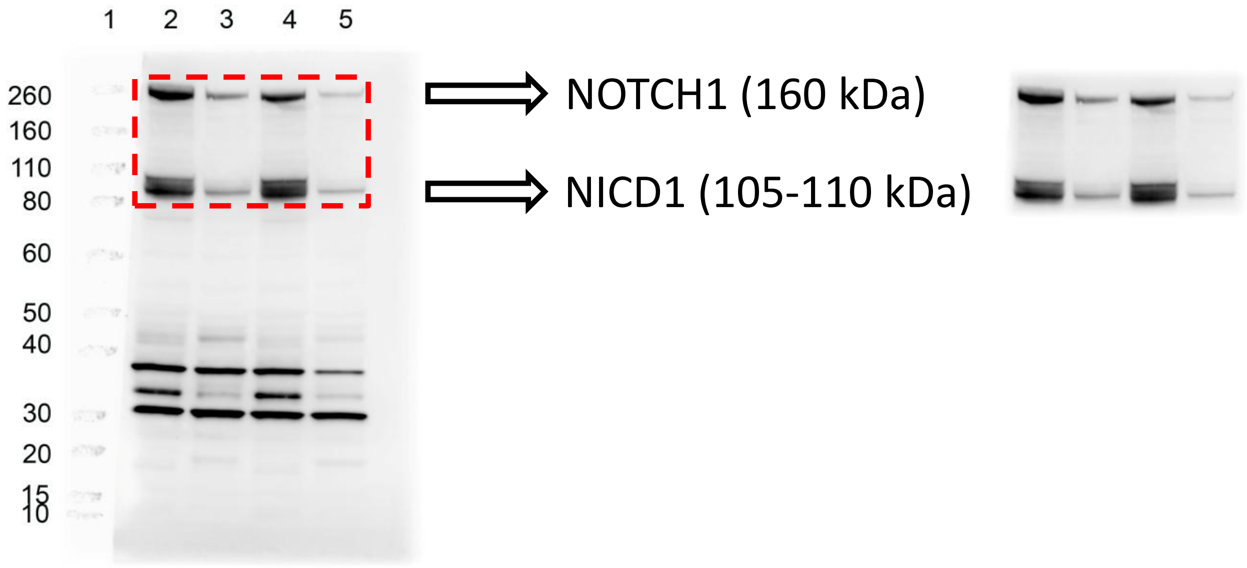
1 2 3 4 5
260
160
110
80
60
50
40
30
20
15
10
NOTCH1 (160 kDa)
NICD1 (105-110 kDa)

b

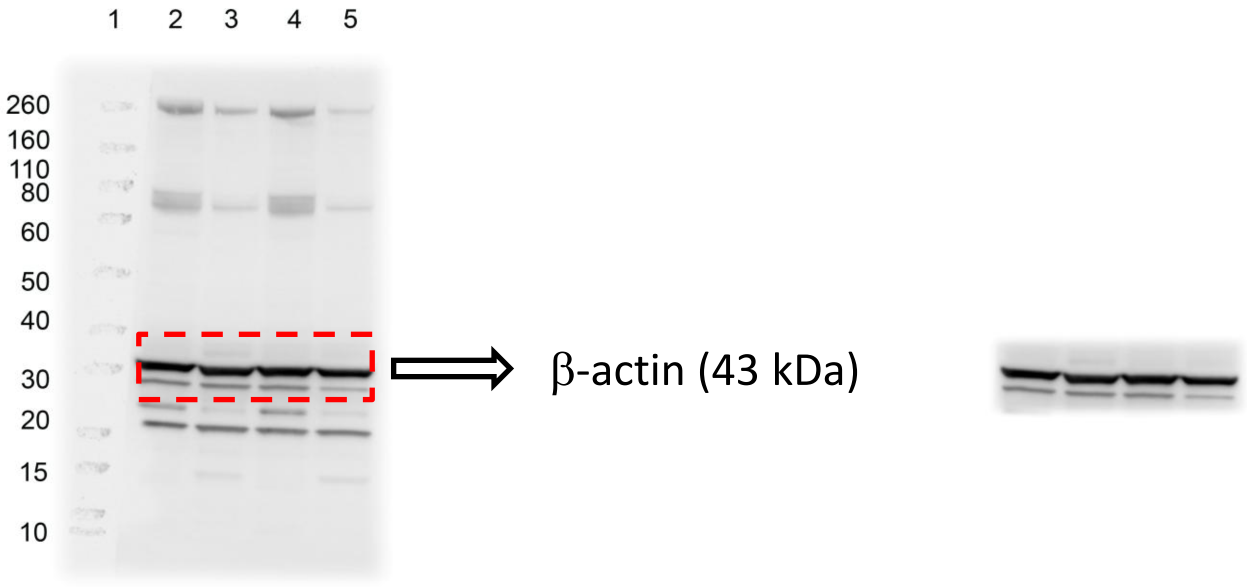
1 2 3 4 5
260
160
110
80
60
50
40
30
20
15
10
β-actin (43 kDa)

S4

a

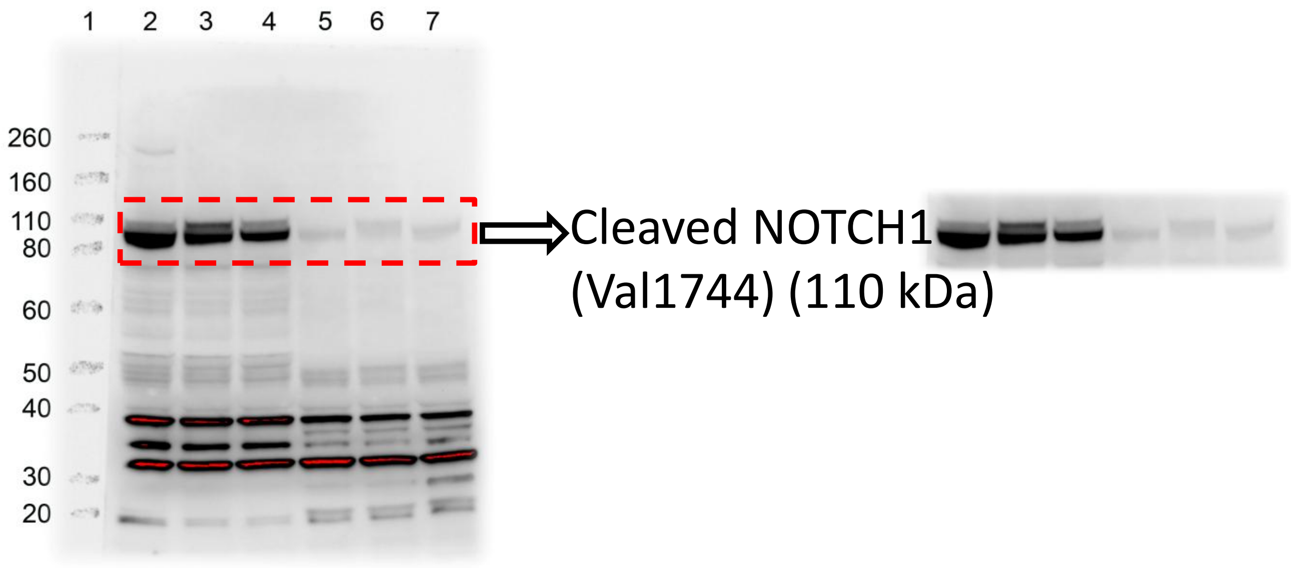
1 2 3 4 5 6 7
260
160
110
80
60
50
40
30
20
Cleaved NOTCH1 (Val1744) (110 kDa)

b

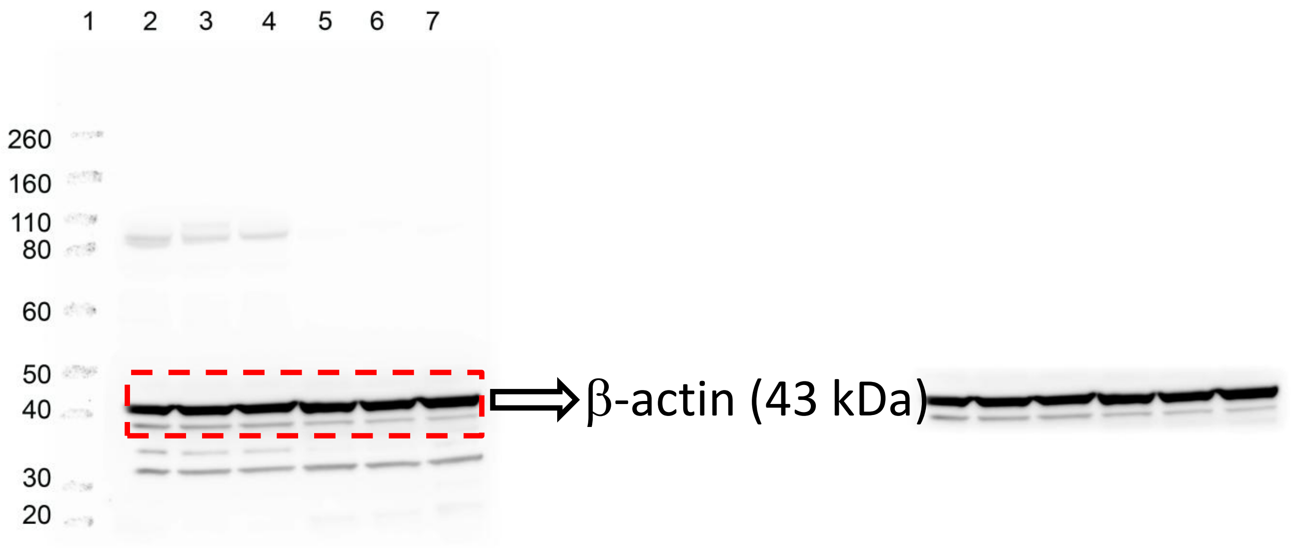
1 2 3 4 5 6 7
260
160
110
80
60
50
40
30
20
β-actin (43 kDa)

S5

a

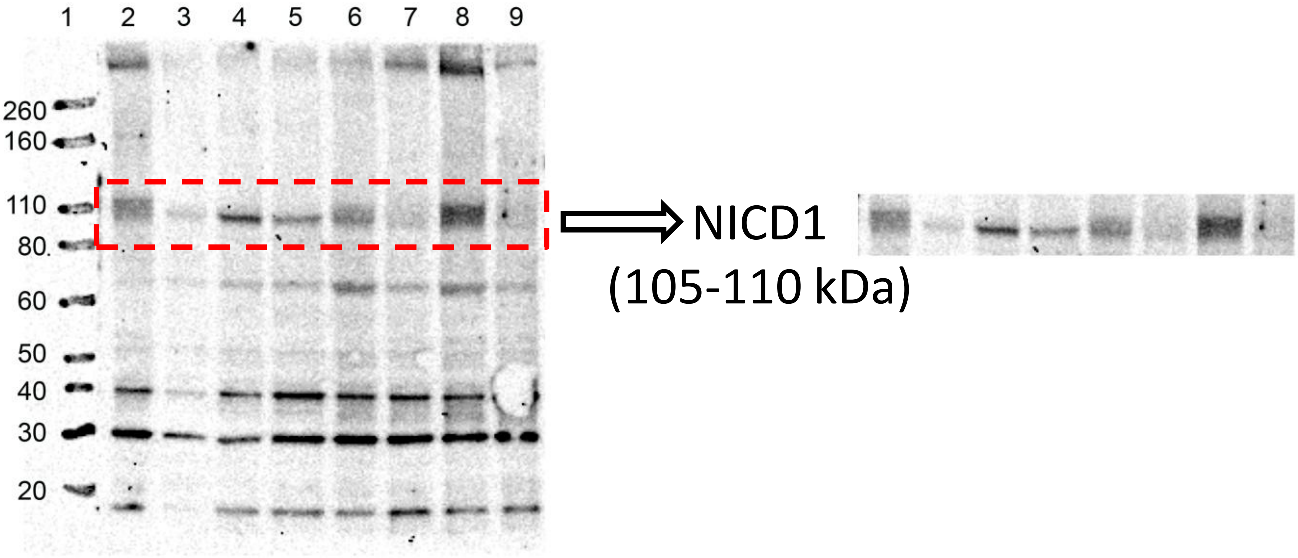
1
2
3
4
5
6
7
8
9
260
160
110
80
60
50
40
30
20
NICD1
(105-110 kDa)

b

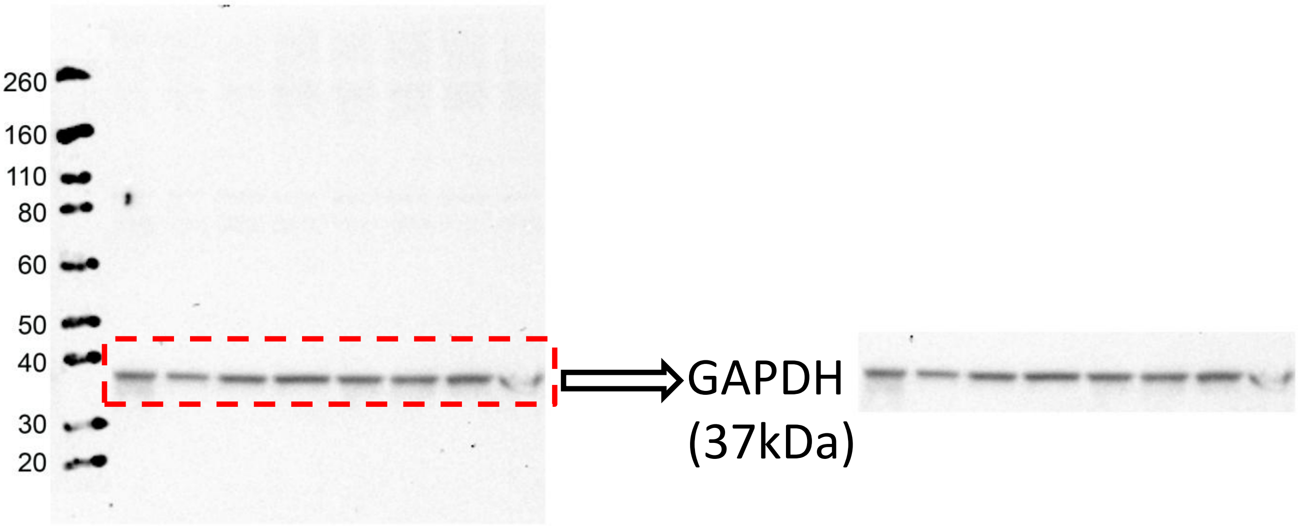
260
160
110
80
60
50
40
30
20
GAPDH
(37kDa)

S6

a

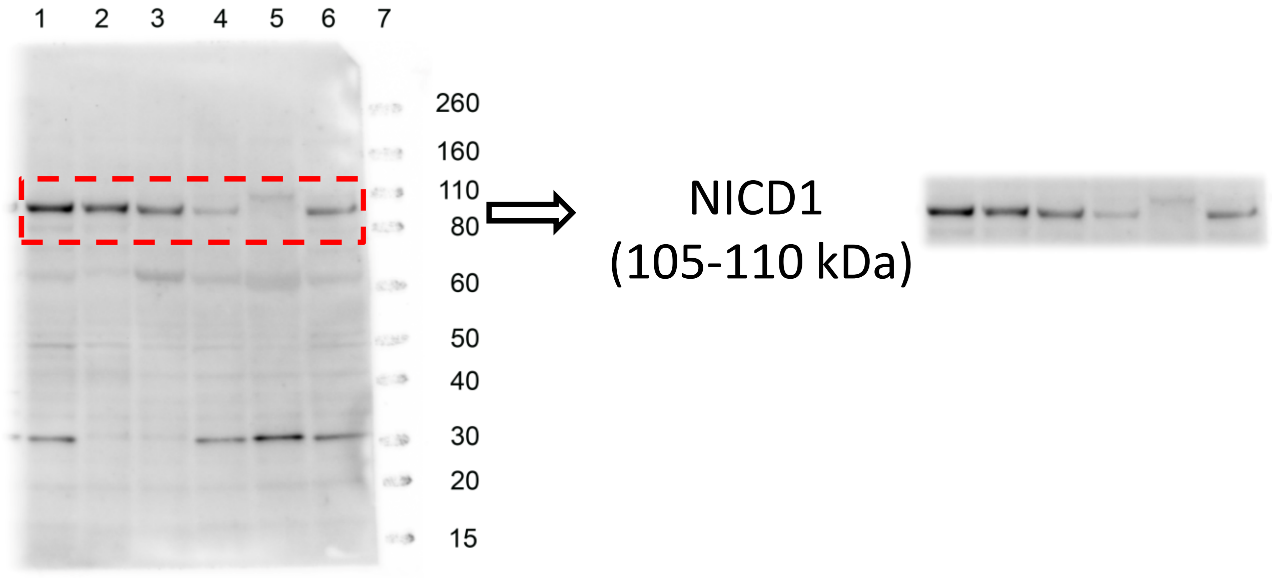
1 2 3 4 5 6 7
260
160
110
80
60
50
40
30
20
15
NICD1
(105-110 kDa)

b

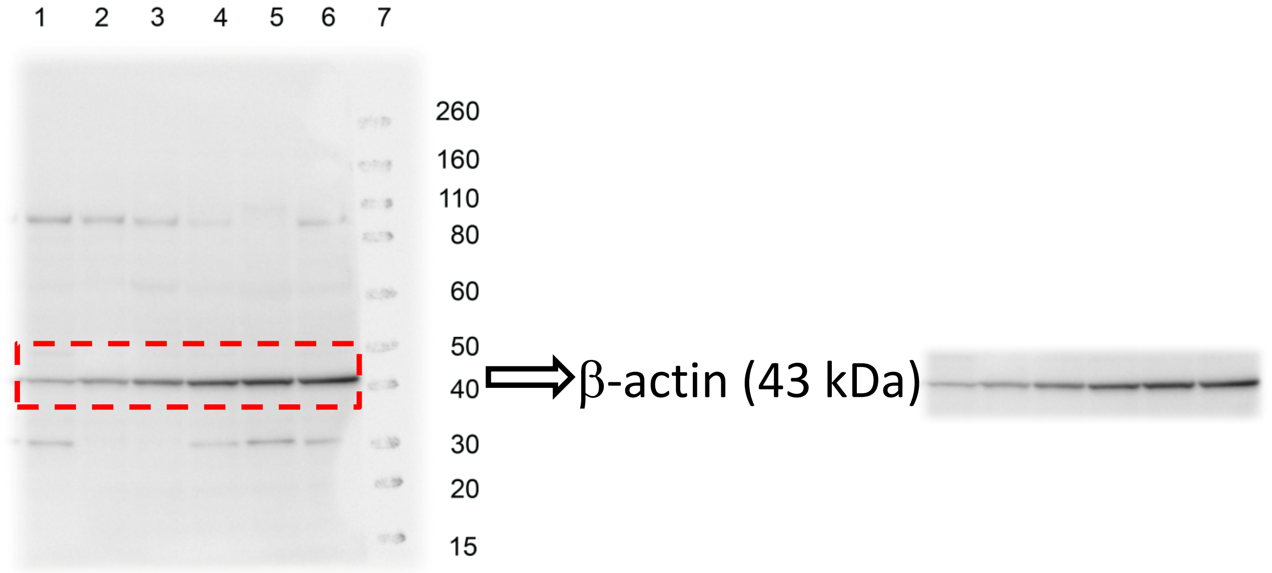
1 2 3 4 5 6 7
260
160
110
80
60
50
40
30
20
15
β-actin (43 kDa)

c

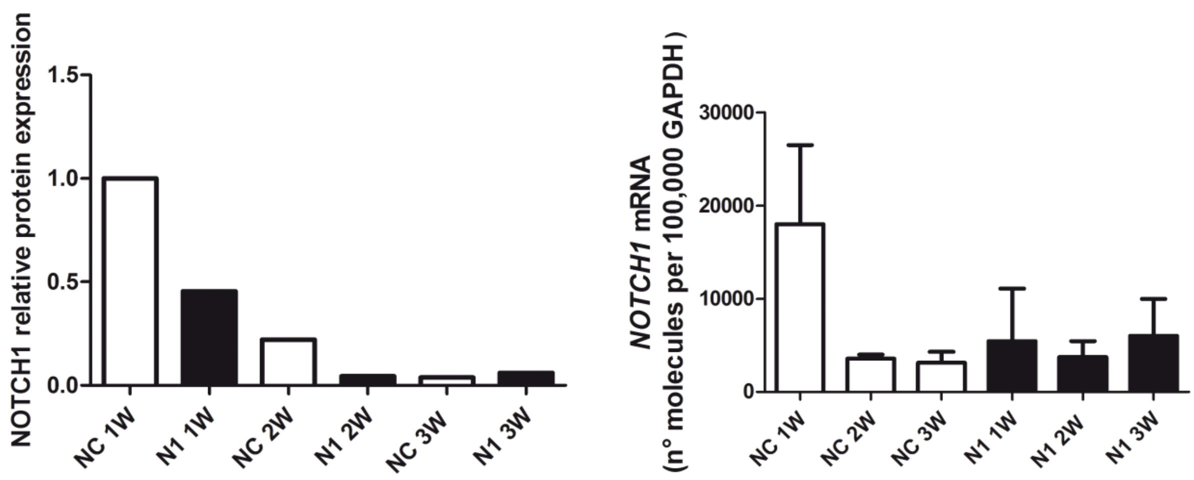
NOTCH1 relative protein expression
1.5
1.0
0.5
0.0
NC 1W
N1 1W
NC 2W
N1 2W
NC 3W
N1 3W
NOTCH1 mRNA
(n° molecules per 100,000 GAPDH)
30000
20000
10000
0
NC 1W
NC 2W
NC 3W
N1 1W
N1 2W
N1 3W

S7

a

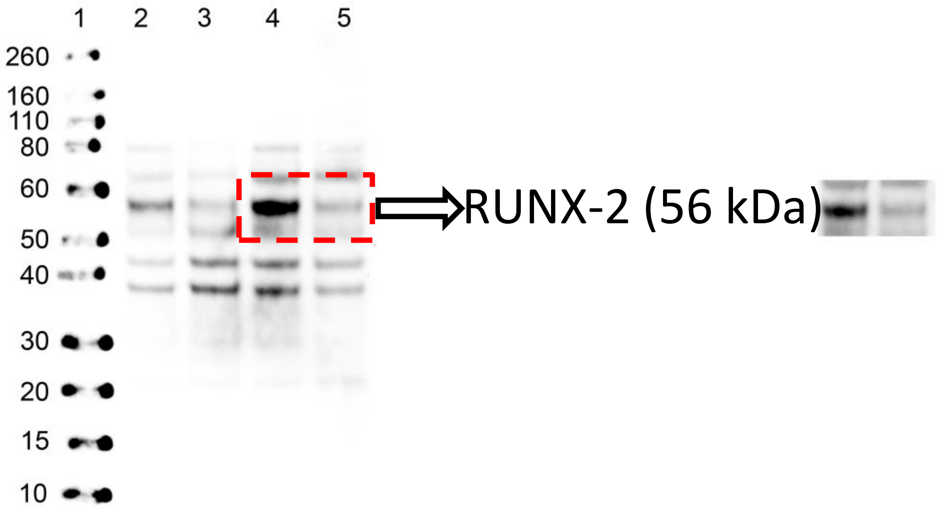
1 2 3 4 5
260
160
110
80
60
50
40
30
20
15
10
RUNX-2 (56 kDa)

b

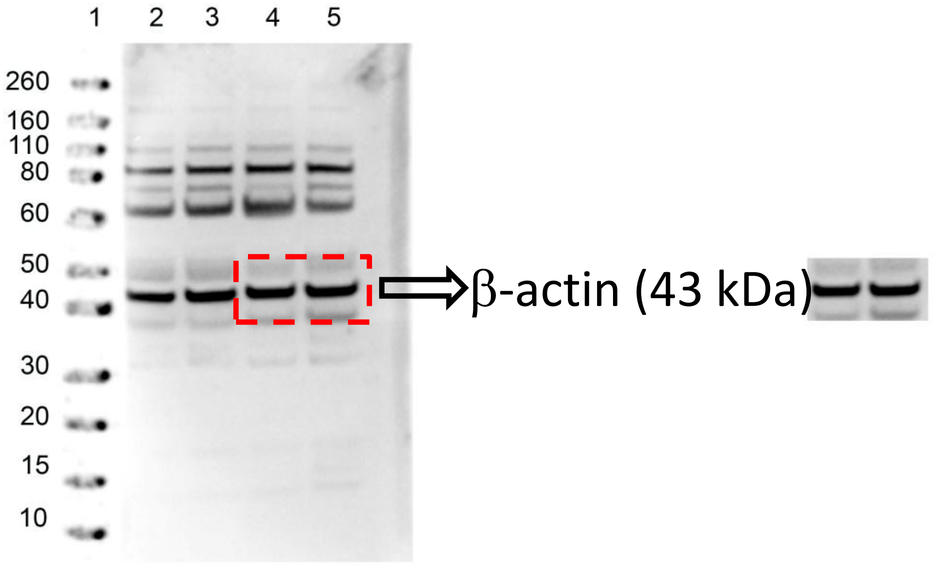
1 2 3 4 5
260
160
110
80
60
50
40
30
20
15
10
β-actin (43 kDa)

S8

a

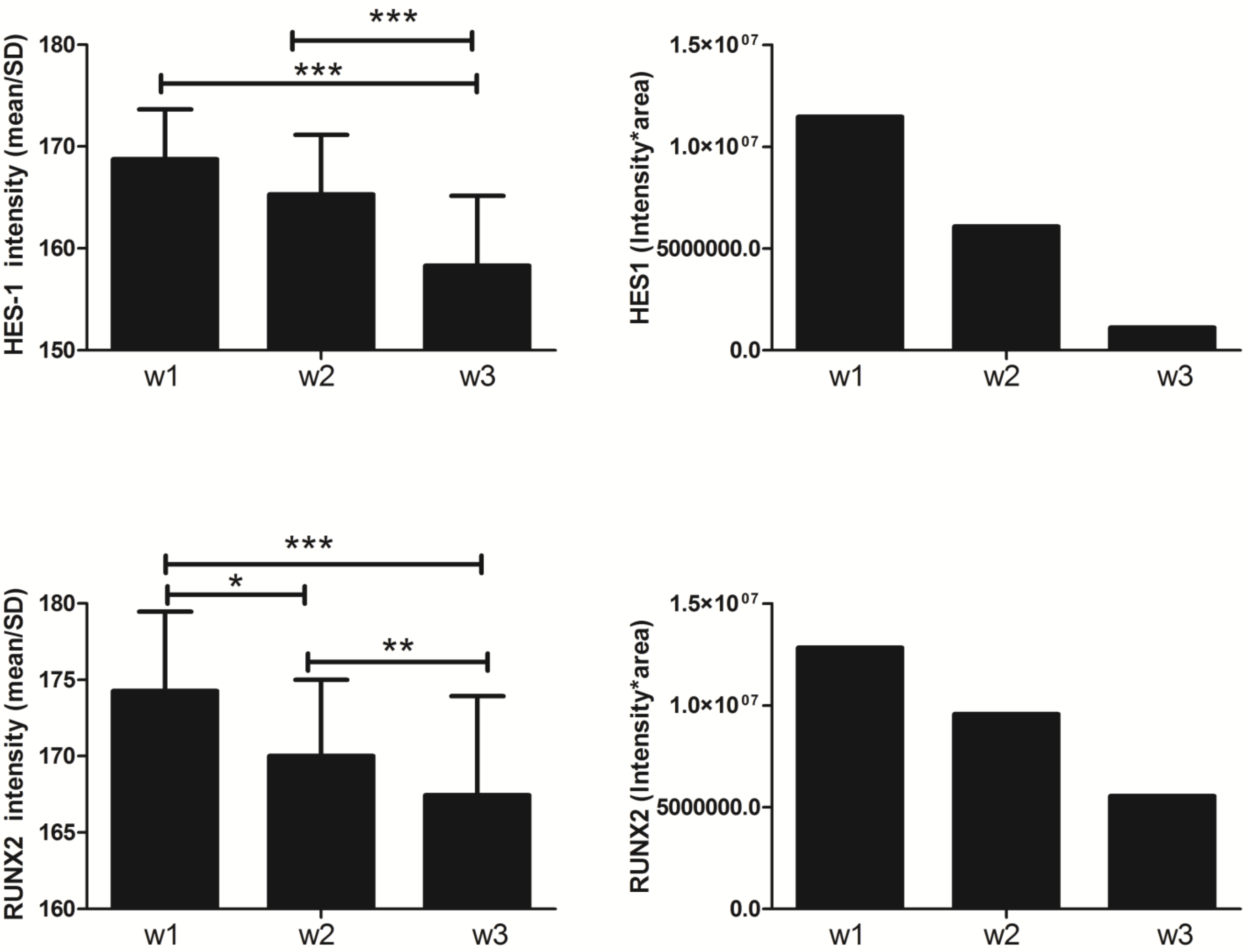

***
***
HES-1 intensity (mean/SD)
180
170
160
150
w1
w2
w3
1.5×10^07
1.0×10^07
5000000.0
0.0
HES1 (Intensity*area)
w1
w2
w3
***
*
**
RUNX2 intensity (mean/SD)
180
175
170
165
160
w1
w2
w3
1.5×10^07
1.0×10^07
5000000.0
0.0
RUNX2 (Intensity*area)
w1
w2
w3

b

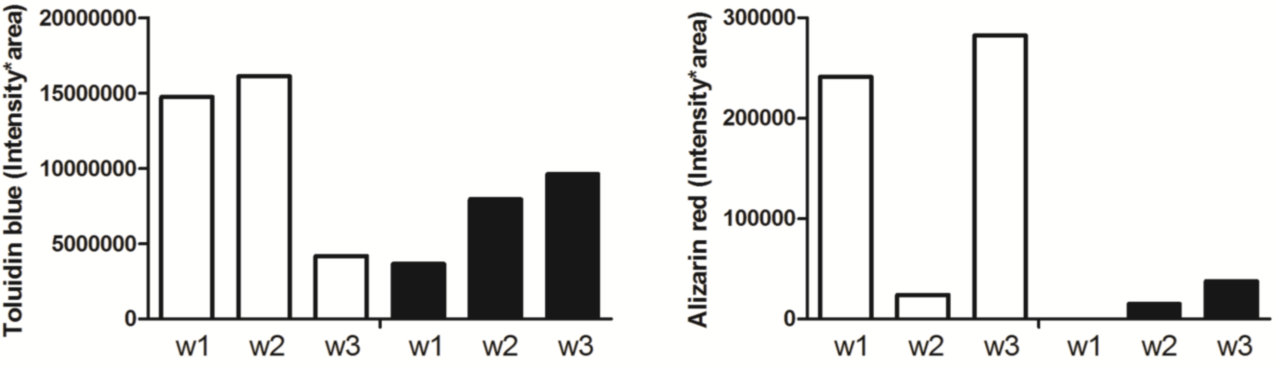

Toluidin blue (Intensity*area)
20000000
15000000
10000000
5000000
0
w1
w2
w3
w1
w2
w3
Alizarin red (Intensity*area)
300000
200000
100000
0
w1
w2
w3
w1
w2
w3

LEGEND TO SUPPLEMENTARY FIGURES

Figure S1: Full blots used to derive the NICD1 and GAPDH results shown in Figure 1a of the main manuscript, obtained with lysates of chondrocytes cultured in either monolayer (2-D) or micromass (3-D) loaded in NuPAGE Novex 4-12% Bis-Tris gels. Samples were run with NuPAGE MES along with Novex Sharp Pre-Stained Protein Standards (lane 1). After protein transfer, the lanes containing the standards were cut from the membranes containing the samples. To assess the molecular weight of western blot stained bands, the pre-stained bands of the markers were highlighted by mean of a Glow Writer pen (http://divbio.com/glow-writerpen.aspx) and at the end of western blotting, the lanes containing the Protein Standards (Novex Sharp Pre-Stained Protein Standard) were juxtaposed to the original membranes. The red dashed rectangles indicate the bands included in the crops, and on the right the arrow indicates the bands corresponding to NICD1 **(a)** and GAPDH **(b)** used to set up Figure 1a. The loading order is as follows: 1) Novex-Sharp Pre-Stained Protein standard; 2) lysate corresponding to half micromass (established with 250,000 cells from patient #1); 3) lysate corresponding to half micromass (established with 250,000 cells from patient #2); 4) lysate corresponding to half micromass (established with 250,000 cells from patient #3); 5) lysate corresponding to 150,000 chondrocytes cultured in monolayer) 6) lysate corresponding to half micromass (established with 250,000 cells from patient #4); 7) lysate corresponding to half micromass (established with 250,000 cells from patient #5); 8) lysate corresponding to half micromass (established with 250,000 cells from patient #6).

Figure S2: Full blots used to derive the NICD1 and β-actin results shown in Figure 1b of the main manuscript, obtained with lysates derived from both C28/I2 immortalized chondrocytes and primary chondrocytes cultured in monolayer and assessed in western blot as previously detailed . The red dashed rectangles indicate the bands included in the crops, and on the right the arrow indicates the bands corresponding to NICD1 **(a)** and β-actin **(b)** used to set up Figure 1b. The marker shown at the left of each image is Novex-Sharp Pre-Stained Protein standard. The loading order is as follows: 1) lysate corresponding to 300,000 C28/I2; 2) lysate corresponding to 300,000 primary chondrocytes; 3) lysate corresponding to 300,000 C28/I2; 4) lysate corresponding to 300,000 primary chondrocytes;

Figure S3: Full blots used to derive the NOTCH1/NICD1 and β-actin results useful to compare NOTCH1/NICD1 intensity in both C28/I2 immortalized chondrocytes and primary chondrocyte cultured at either low density (LD) or high density (HD). Western blot was performed as detailed above. The red dashed rectangles indicate the bands included in the crops, and on the right the arrow indicates the bands corresponding to

NOTCH/NICD1 **(a)** and β-actin **(b)** for densitometric analysis. Densitometric analysis confirms that HD cultures have less NOTCH1/NICD1 compared to LD cultures and that in both conditions the signal relative to primary chondrocytes is about 17% compared to C28/I2 cells. NOTCH1/NICD1 signal is reduced at high density down to about 45% (although the number of cells loaded at high density is 20% higher). The loading order is as follows: 1) Novex-Sharp Pre-Stained Protein standard; 2) lysate corresponding to 200,000 C28/I2 LD; 3) lysate corresponding to 200,000 primary chondrocytes LD; 2) lysate corresponding to 250,000 C28/I2 HD; 3) lysate corresponding to 250,000 primary chondrocytes HD.

Figure S4: Full blots used to compare the signal of Cleaved NOTCH1 (Val1744) in C28/I2 immortalized chondrocytes or primary chondrocytes in basal conditions or after 1 hour stimulation with either 5 mM EDTA or 2.5 ng/ml IL-1β. β-actin was used as a loading control. Western blot was performed as detailed above. The red dashed rectangles indicate the bands included in the crops, and on the right the arrow indicates the bands corresponding to Cleaved NOTCH1 (Val1744) **(a)** and β-actin **(b)**. The loading order is as follows: 1) Novex-Sharp Pre-Stained Protein standard; 2) lysate corresponding to 250,000 C28/I2 in basal conditions; 3) lysate corresponding to 250,000 C28/I2 after 1 hour treatment with 5mM EDTA; 4) lysate corresponding to 250,000 C28/I2 after 1 hour treatment with 2.5 ng/ml IL-1β; 5) lysate corresponding to 250,000 primary chondrocytes in basal conditions; 6) lysate corresponding to 250,000 primary chondrocytes after 1 hour treatment with 5mM EDTA; 7) lysate corresponding to 250,000 primary chondrocytes after 1 hour treatment with 2.5 ng/ml IL-1β.

Figure S5: Full blots used to derive the NICD1 and GAPDH results shown in Figure 2a of the main manuscript, obtained with lysates of chondrocytes in either control (NC, control siRNA) or NOTCH-1 KD (N1, NOTCH1 siRNA) conditions, loaded in NuPAGE Novex 4-12% Bis-Tris gels. Samples were run with NuPAGE MES along with Novex Sharp Pre-Stained Protein Standards. Western blot was performed as detailed above. The red dashed rectangles indicate the bands included in the crops, and on the right the arrow indicates the bands corresponding to NICD1 **(a)** and GAPDH **(b)** used to set up Figure 2a. The loading order is as follows: 1) Novex-Sharp Pre-Stained Protein standard; 2) lysate corresponding to 150,000 control (NC) chondrocytes from patient #1; 3) lysate corresponding to 150,000 NOTCH-1 KD (N1) chondrocytes from patient #1; 4) lysate corresponding to 150,000 control (NC) chondrocytes from patient #2; 5) lysate corresponding to 150,000 NOTCH-1 KD (N1) chondrocytes from patient #2; 6) lysate corresponding to 150,000 control (NC) chondrocytes from patient #3; 7) lysate corresponding to 150,000 NOTCH-1 KD (N1) chondrocytes from patient #3); 8) lysate

corresponding to 150,000 control (NC) chondrocytes from patient #4; 9) lysate corresponding to 150,000 NOTCH-1 KD (N1) chondrocytes from patient #4.

Figure S6: **(a)** and **(b): f**ull blots used to derive the NICD1 and β-actin results useful to assess the persistence of NOTCH1 silencing at the protein level across 1, 2 and 3 week maturation of micromasses obtained with chondrocytes in either control (NC, control siRNA) or NOTCH-1 KD (N1, NOTCH1 siRNA) conditions from one representative patient. Lysates corresponding to one half micromass in each condition were loaded in NuPAGE Novex 4-12% Bis-Tris gels. Samples were run with NuPAGE MES along with Novex Sharp Pre-Stained Protein Standards. Western blot was performed as detailed above. The red dashed rectangles indicate the bands included in the crops, and on the right the arrow indicates the bands corresponding to NICD1 **(a)** and β-actin **(b)** used for densitometric analysis whose results are presented in the left graph of Figure **(c)**. In addition, the right graph in **(c)** shows the level of NOTCH1 RNA across 1, 2 and 3 weeks maturation derived from 4 different patients, expressed as number of molecules per 100,000 GAPDH molecules with mean±SD. The loading order is as follows: 1) lysate corresponding to one half NC micromass control at 1 week maturation; 2) lysate corresponding to one half N1 micromass control at 1 week maturation; 3) lysate corresponding to one half NC micromass at 2 week maturation; 4) lysate corresponding to one half N1 micromass at 2 week maturation; 5) lysate corresponding to one half NC micromass at 3 week maturation; 6) lysate corresponding to one half N1 micromass control at 3 week maturation; 7) Novex-Sharp Pre-Stained Protein standard.

Figure S7: Full blots used to derive the RUNX-2 and β-actin results shown in Figure 5 of the main manuscript, obtained with lysates of chondrocytes cultured in 3-D, in either control (NC, control siRNA) or NOTCH-1 KD (N1, NOTCH1 siRNA) conditions, loaded in NuPAGE Novex 4-12% Bis-Tris gels. Samples were run with NuPAGE MES along with Novex Sharp Pre-Stained Protein Standards. Western blot was performed as detailed above. The red dashed rectangles indicate the bands included in the crops, and on the right the arrow indicates the bands corresponding to RUNX-2 **(a)** and β-actin **(b)** used to set up Figure 5. The loading order is as follows: 1) Novex-Sharp Pre-Stained Protein standard; 2) lysate corresponding to 1 micromass established with control (NC) chondrocytes from patient A; 3) lysate corresponding to 1 micromass established with NOTCH-1 KD (N1) chondrocytes from patient A; 4) lysate corresponding to 1 micromass established with control (NC) chondrocytes from patient B; 5) lysate corresponding to 1 micromass established with NOTCH-1 KD (N1) chondrocytes from patient B.

Figure S8: image analysis of the immunohistochemistry, toluidin blue and alizarin red results shown in Figure 5. A quantification of the images shown in Figure 5 was

performed exploiting the Nikon Imaging Software (NIS) and the data obtained were graphically represented in Supplementary Figure 8. For each signal, tissue areas underwent a thresholding using the intensity function of the software. The thresholds were as follows: threshold of 100-155 for immunohistochemistry to evaluate HES1 and RUNX2 signals and also for toluidin blue, and threshold of 15-36 for alizarin red. NIS identified several areas (from tens to hundreds for each section with more areas in section with higher staining) and produced an output with several calculated parameters, including mean intensity and area for each. These data were used in a statistical analysis to compare the conditions. We performed ANOVA and found the statistical significances reported in Supplementary Figure 8 (a) in left graphs. To emphasize the information that the cumulative area identified by NIS as "beyond the threshold" was variable according to the specific condition, we also included the graphical representation of the product of the mean intensity and the cumulative area (Figure S8a, right graphs). This better represents the trend of the signal, but we could only perform the statistical comparison on the left graphs. For toluidin blue and alizarin red we only reported the intensity per area evaluation, since in some cases no staining was detectable (Supplementary Figure 8b).
